# Investigating the Impact of Flavonoids on *Aspergillus flavus*: Insights into Cell Wall Damage and Biofilms

**DOI:** 10.3390/jof10090665

**Published:** 2024-09-23

**Authors:** Lina Castano-Duque, Matthew D. Lebar, Brian M. Mack, Jessica M. Lohmar, Carol Carter-Wientjes

**Affiliations:** United States Department of Agriculture—Agriculture Research Services, New Orleans, LA 70124, USA; matthew.lebar@usda.gov (M.D.L.); brian.mack@usda.gov (B.M.M.); jessica.lohmar@usda.gov (J.M.L.); carol.carter@usda.gov (C.C.-W.)

**Keywords:** *Aspergillus*, aflatoxin, quercetin, luteolin, apigenin, cell wall

## Abstract

*Aspergillus flavus*, a fungus known for producing aflatoxins, poses significant threats to agriculture and global health. Flavonoids, plant-derived compounds, inhibit *A. flavus* proliferation and mitigate aflatoxin production, although the precise molecular and physical mechanisms underlying these effects remain poorly understood. In this study, we investigated three flavonoids—apigenin, luteolin, and quercetin—applied to *A. flavus* NRRL 3357. We determined the following: (1) glycosylated luteolin led to a 10% reduction in maximum fungal growth capacity; (2) quercetin affected cell wall integrity by triggering extreme mycelial collapse, while apigenin and luteolin caused peeling of the outer layer of cell wall; (3) luteolin exhibited the highest antioxidant capacity in the environment compared to apigenin and quercetin; (4) osmotic stress assays did not reveal morphological defects; (5) flavonoids promoted cell adherence, a precursor for biofilm formation; and (6) RNA sequencing analysis revealed that flavonoids impact expression of putative cell wall and plasma membrane biosynthesis genes. Our findings suggest that the differential effects of quercetin, luteolin, and apigenin on membrane integrity and biofilm formation may be driven by their interactions with fungal cell walls. These insights may inform the development of novel antifungal additives or plant breeding strategies focusing on plant-derived compounds in crop protection.

## 1. Introduction

*Aspergillus flavus* is an opportunistic plant pathogen capable of infecting economically important oil seed crops such as peanuts, corn, and cotton and further contaminating them with a group of polyketide-derived mycotoxins known as aflatoxins [1]. Aflatoxins are notoriously toxic and carcinogenic mycotoxins, which are strictly regulated in food and feed [1]. Currently, 20 different aflatoxins have been described, with aflatoxin B1 being characterized as the most carcinogenic [2,3]. Ingesting high concentrations of aflatoxins causes acute toxicity, resulting in severe illness and death, while long-term chronic low-dose exposure to aflatoxins leads to the development of liver cancer [4,5,6]. Regulation of aflatoxins in food and feed affords consumer safety; however, contamination still results in significant economic losses [2,7]. Globally, mycotoxins are estimated to affect one-quarter of crops each year, with aflatoxins regarded as the most important due to their carcinogenic properties [8]. Preventing *A. flavus* from infecting crops or mitigating its ability to produce aflatoxins would result in a safer, more affordable food supply.

Aflatoxin contamination of oilseed crops grown for food and feed, particularly corn, is of great concern [1]. Climate change is capable of making plants more susceptible to fungal infections and diseases [9]. This will likely lead to even more contamination and exacerbate monetary losses for the corn industry. Potential economic losses due to aflatoxin contamination of corn are estimated to be greater than USD 1.6 billion per year in the United States if global warming continues [7]. Corn plants that are resistant to aflatoxin contamination would reduce economic losses and improve global health. We previously assessed transcriptomes of corn lines resistant (TZAR102 and MI82) to aflatoxin contamination to probe the molecular mechanisms of resistance [10]. We found that higher flavonoid levels in corn kernels correlated with a lower proliferation of *A. flavus* and subsequently decreased aflatoxin toxin production. Flavonoids are a diverse group of secondary metabolites that naturally occur in many plants and have a broad range of functions, including chemical messaging and alleviating physical stress [11]. These compounds show promise as antifungal agents [12] and have been proposed as alternatives and/or supplements to conventional fungicides [13]. Also, in plants, flavonoids show anti-microbial and antifungal properties that are part of the constitutive plant defense mechanisms [14,15], modulation of plant–microbial interactions, and overall regulation of the plant–root microbiome [16]. Flavonoids are differentiated by their chemical structures as follows: flavones (apigenin), flavanones (naringenin), flavans (catechin), flavonoid glycosides, flavonols (quercetin), flavonolignan (silibinin), chalcones (butein), isoflavones (genistein), aurones (aureusidin), leucoanthocynidins (leucopelargonidin), and neoflavonoids (neoflavones) [17]. The mechanisms by which flavonoids affect fungal growth and aflatoxin production are complicated and require further investigation.

The modulation of fungal communities by flavonoids has been explored previously from root exudates and soil microbes [18,19]. There is a feedback loop in which plants increase flavonoid production under fungal infections [10,20], and the soil–root microbiome is influenced by flavonoid production in roots [20,21,22]. Flavonoids can mediate communication between plants and plant-growth-promoting bacteria. Biosynthesis of flavonoids in rice can increase resistance to pathogenic bacteria [18]. Plant–microbe interactions are mediated by flavonoids; Wang et al. [16] reviewed several examples of these interactions, e.g., in the *Fabaceae* family, including that *Rhizobium* nodulation is modulated by flavonoids. During pathogenic interactions, sakuranetin (flavanone) in rice increased resistance to *Fusarium fujikuroi*, *Magnaporthe oryzae*, and *Rhizoctonia solani*. In sorghum, luteolin is toxic to spores of *Colletotrichum sublineola*, and transgenic sorghum that produces flavonols and anthocyanidins is resistant to *C. sublineola*. Transgenic *Medicago truncatula*, which produces fewer flavonoids, is more sensitive to *Fusarium oxysporum* infection. Also, transgenic lines of barley that produce fewer flavonoids have higher incidences of infection by *Fusarium graminearum* [16]. Microbiome experiments conducted using corn seedlings showed that mutant lines with insertions in the benzoxazinoid pathway showed a correlation between BX-controlled root metabolites, particularly flavonoids, and constriction of the soil microbial taxa and stimulation of methylophilic bacteria [23]. The underlying molecular mechanism of flavonoid modulation of pathogen interactions is not fully understood. Górniak, Bartoszewski, and Króliczewski [24] reviewed these mechanisms and concluded that in most cases, these molecules act as antimicrobial compounds that lyse or rupture the membrane as well as inhibit biofilm formation, nucleic acid synthesis, electron transport, and ATP synthesis. Initial investigations showed that simple flavones had differential effects on mycelial growth in *A. flavus* at high concentrations [25]. Unsubstituted flavone inhibited mycelial growth up to 70%, while 3-hydroxyflavone increased growth by about 10% at high micromolar concentrations (50–800 µM). Anthocyanidins and related flavonoids were found to inhibit aflatoxin B1 biosynthesis in *A. flavus* NRRL 3357 at high concentrations (0.3–9.7 mM) [26].

Previously, we found that low concentrations of flavonoids have a variety of effects on *A. flavus* [27]. They can modulate the proliferation of the fungus as well as damage the cell wall. Vesicle-like structures containing flavonoids formed in *A. flavus* mycelia when treated with low concentrations of flavonoids. Low concentrations of flavonoids were also shown to decrease aflatoxin accumulation. Herein, we expand our investigation of the physical effects and molecular mechanisms of several flavonoids on *A. flavus.* We assess the physical structure of mycelia, ergosterol production and localization, osmotic stress response, and expression of cell wall biosynthesis genes in *A. flavus* NRRL 3357.

## 2. Materials and Methods

### 2.1. Bioassays

One *A. flavus* strain was used, NRRL 3357, as described before [28], herein called AF3357. Flavonoids (apigenin, luteolin, luteolin-7-glucoside, and quercetin) were purchased from Indofine Chemical Co. (Hillsborough Township, NJ, USA). To 50 mL Erlenmeyer flasks, we added 40 mL of 1 × 10^4^ CFU/mL fungal inoculum in potato dextrose broth (DIFCO^TM^ PDB—Becton, Dickison and Co., Sparks, MD, USA) with 0.0001% Triton-X (pH 6.0) [29,30]. To the inoculated media, we added each of the flavonoid treatments at various concentrations of weights to final volumes (0, 0.001, 0.0001, and 0.00001 µg/µL) that were dissolved in dimethyl sulfoxide (DMSO, J.T. Baker, Phillipsburg, NJ, USA). The flavonoid stock solutions were prepared in 100% DMSO prior to adding them to 40 mL of fungal inoculum, making the final concentration 1% DMSO in 40 mL. The concentrations used for these compounds were selected based on their aflatoxin-inhibitory capacity described in previous studies [27] and their solubility in 100% DMSO and after adding them to the fungal inoculum. Both flavonoid treatments and controls were exposed to the same concentration of DMSO (1%, 140 mM). Four replicates per flavonoid were used, with the DMSO control containing 1% DMSO or control without DMSO. Flasks were incubated at 31 °C in darkness with 90 RPM shaking, and experiments were carried out separately for 16, 24, and 72 h. At each time point, mycelia and media were separated by filtering the mycelia using a 5 µm cell strainer (pluriSelect, El Cajon, CA, USA). Sub-samples of mycelia were fixed by adding glutaraldehyde to a final 4% concentration in a 1% phosphate buffer and prepared for microscopy analysis.

### 2.2. Corn-Enriched Plate Bioassay

AF3357 (1 × 10^4^ CFU/mL initial concentration in 0.0001% Triton-X) was single-point inoculated in PDA plates enriched with corn meal powder. Corn-enriched plates were made using a 60%/40% (*w*/*w* ratio) ratio of PDA to corn powder from the B73 corn genotype. Prior to single-point inoculation, 500 µL of 0.001 µg/µL of flavonoids (apigenin, luteolin, luteolin-7-glucoside, and quercetin) or 1% DMSO or buffer control (*n* = 4) were spread on the surface of the plate and allowed to be absorbed into the agar. Plates were incubated at 31 °C in darkness for 6 to 7 days, and colony diameter was measured daily.

### 2.3. Biofilm Adherence Assay

AF3357 (1 × 10^4^ CFU/mL initial concentration into PDB medium (pH 6.0) with 0.0001% Triton-X) treated with 0.001 µg/µL of flavonoids (apigenin, luteolin, luteolin-7-glucoside, and quercetin) and two controls (1% DMSO or buffer control; *n* = 4) were incubated for 72 h at 31 °C in darkness in 24-well plates (volume per well of 2 mL) [31]. After incubation, the mycelial mat on the surface of the well and the media were removed, leaving only the mycelia that adhered to the walls of the wells. Media were removed from each well, and then 2 mL of 0.01% crystal violet (dissolved in water) was added to each well and incubated for 20 min at room temperature. The supernatant was removed and allowed to dry, the wells were de-stained with 2 mL of 30% acetic acid, and the OD was measured at 560 nm.

### 2.4. Antioxidant Assay

AF3357 (1 × 10^4^ CFU/mL initial concentration into PDB medium (pH 6.0) with 0.0001% Triton-X) with various flavonoid concentrations (0.001, 0.0001, and 0.00001 µg/µL) and 1% DMSO (*n* = 4) was incubated for 72 h at 31 °C in darkness. Antioxidant capacity was measured following the manufacturer’s instructions (DPPH Antioxidant assay kit, Dojindo, Japan).

### 2.5. Osmotic Stress Assays

Exposure of flavonoid-treated mycelium to osmotic stress agents was carried out utilizing methods similar to those in Baidya et al. [32] with minor modifications. Briefly, the AF3357 strain was inoculated and incubated in the same manner as utilized in the adherence assay as described above. After 72 h of incubation, mycelium was harvested from each treatment and macerated into 10 mL of 1% PBS. A total of 10 µL of each macerated culture was transferred onto 10 mL of PDA medium and PDA medium containing either 0.6 M KCl or 1 M sorbitol in triplicate. The cultures were incubated at 31 °C under dark conditions for 72 h prior to observing the cultures for morphological defects and then photographed.

### 2.6. Microscopy

Bright-field images and fluorescence images were taken using an SMZ25 stereoscope (Nikon Metrology Inc., Brighton, MI, USA) and an upright Eclipse Ni-E microscope (Nikon Metrology Inc.) as previously described by Rajasekaran et al. [33]. Scanning electron microscopy (SEM) was conducted on a HITACHI S-4800 FEG CRYO-SEM (Hitachi High-Tech America, Inc., Los Angeles, CA, USA). For SEM analysis, AF3357 was incubated with apigenin, luteolin, and quercetin (0.001 µg/µL) for 72 h at 31 °C in darkness (1 × 10^4^ CFU/mL initial concentration into PDB (pH 6.0) with 0.0001% Triton-X). After incubation, the samples were fixed with 4% glutaraldehyde. Fixed samples were dehydrated by passage through increasing concentrations of ethanol solutions (25–100%) and then critical-point dried and coated with graphite (usually 100 or 200 Å).

### 2.7. Filipin Staining

Glutaraldehyde-fixed mycelia were stained by using a filipin complex (Sigma-Aldrich, St. Louis, MO, USA) that binds to ergosterol. Mycelia were rinsed with 1% phosphate buffer three times, then incubated for 10 min with 1.5 mg/mL glycine in 1% PBS, rinsed with 1% PBS, and incubated with filipin working solution (0.005 mg/mL or 15 µM) for 2 h at room temperature. Samples were rinsed with 1% phosphate buffer and mounted with 40 µL of anti-fade reagent (ProLong^TM^ Gold Antifade Reagent—Invitrogen, Eugene, OR, USA) to prevent excessive photobleaching. Samples were visualized using the fluorescence filter for 4′-6-diamidino-2-phenylindole (DAPI—excitation of 358 nm and emission of 461 nm).

### 2.8. Naturstoff Reagent A (DPBA) Staining for Flavonoid Staining

We used a DPBA (diphenylboric acid 2-aminoethyl ester) assay to determine flavonoid localization in the fungi as previously described [27]. Briefly, AF3357 was incubated for 72 h at 31 °C in darkness (1 × 10^4^ CFU/mL initial concentration into PDB medium (pH 6.0) with 0.0001% Triton-X). Mycelia were removed and fixed with 4% glutaraldehyde in 1X PBS. Then, 50 µL of 0.5% (*w*/*v*) DPBA (Sigma D9754-5G) in methanol was added to each well for flavonoid staining [34]. After 1 h of incubation at room temperature with shaking, the mycelia were removed from the well and visualized using fluorescence settings in an upright Eclipse Ni-E microscope (Nikon Metrology Inc., Brighton, MI, USA).

### 2.9. Ergosterol Analysis

The filtered mycelia were separately lyophilized for ergosterol analysis, and dry mycelia weights were recorded. Alcoholic potassium hydroxide (KOH) was prepared: 125 g KOH was dissolved in 175 mL water, and then 100% ethanol was added for total volume of 500 mL. Ten milliliters of the alcoholic KOH solution was added to each lyophilized mycelium sample, and the mixture was vortexed for 30 s and then incubated at 85 °C in a water bath for 1.5 h. The samples were then cooled to room temperature, diluted with 3.3 mL of water, and extracted with hexanes (10 mL). The hexanes layers were carefully transferred to clean scintillation vials and concentrated via speedvac (Savant, Thermo Scientific, Waltham, MA, USA). The concentrated extracts were redissolved in methanol (1.5 mL) for analysis. The redissolved solution was analyzed (1 µL injections) using a Waters ACQUITY UPLC system (Waters Corporation, Milford, MA, USA) (95% methanol in water, BEH C18 1.7 μm, 2.1 mm × 50 mm column) with UV detection (λ = 282 nm). An analytical standard of ergosterol (Sigma-Aldrich, St. Louis, MO, USA) was used for quantification. Ergosterol content is reported as µg/g mycelia dry wt.

### 2.10. Single Gene Expression

Fungal samples (*n* = 3) were ground by using 800 µm zirconium beads (OPS diagnostics, Lebanon, NJ, USA) and a bullet blender (Next Advance, Inc., Troy, NY, USA) for 30 s at 6000 strokes/min. Sample RNA was extracted using Zymo^®^ following the manufacturer’s instructions. RNA content was measured using a Nanodrop (ThermoFisher Scientific), and cDNA was made using an iScript cDNA synthesis Kit (Bio-Rad, Hercules, CA, USA) following the manufacturer’s instructions. Reverse transcription quantitative PCR (RT-qPCR) analyses were conducted using Beta-tubulin (*F9C07_8286; AFLA_008802*) for the endogenous control (F: GACACCGTTGTTGAGCCCTA; R: GTCACCGTAAGAGGGGTTGG) and the following target genes (Table S1): *flbA* (conidiophore biogenesis), *scIR* (branching and intertwined aerial hyphae), *gls2* (glucan synthase), chitin synthase, and *Mp1* (cell wall mannoprotein1). The PCR conditions used were 95 °C for 10 min, then 60 cycles of 95 °C for 15 s, 60 °C for 30 s, and 72 °C for 20 s followed by cooling. The relative quantification values were obtained by using the LightCycler^®^ 480 software (version 1.5.1.62; Roche, Basel, Switzerland).

### 2.11. RNA-Seq

Fungal samples (*n* = 3) from cell adhesion bioassays treated with 0.001 µg/µL of flavonoids (apigenin, luteolin, luteolin-7-glucoside, and quercetin), 1% DMSO, or buffer control were sent to Omega Biosciences (Norcross, GA, USA) for sequencing. RNA was sequenced using an Illumina HiSeq 2500 machine, generating an average of 39 million 150 bp paired-end reads per sample. Adapters and low-quality sequences were removed from the reads using fastp [35] (version 0.23.3). Trimmed reads were aligned to the *A. flavus* NRRL 3357 genome (JCVI-afl1-v2.0; GCA_000006275.2) using STAR (version 2.7.10b) with the following settings “—alignIntronMax 2000—twopassMode Basic”. Reads mapping to genes were counted using featureCounts [36] (version 2.04) with the settings: “-p -s 2—primary—countReadPairs”. To identify genes that were differentially expressed between the treatments and the DMSO control, we used the DESeq2 R package (version 1.36.0) [37]. We applied the lfcShrink function with the options type “ashr” [38] and alpha = 0.05 to estimate the log2 fold changes and their *p*-values. The results were then filtered to keep only genes that had an adjusted *p*-value < 0.05 and an absolute log2 fold change > 1. Functional term enrichment of the differentially expressed genes was conducted using the enrichment function in the BC3NET R package [39] (version 1.0.4), which uses Fisher’s one-sided exact test. The false discovery rate was controlled using the p.adjust R function with the method set to “fdr” [40]. To generate the PCA figure, regularized log counts were produced with DESeq2′s rlog function with the option “blind = TRUE” and used as inputs to the plotPCA function in DESeq2, which used the top 500 most variable genes.

### 2.12. Analysis of Gene Expression of Putative Galactosaminogalactan (GAG)-Related Genes in A. flavus

Heatmaps were made using the tidyHeatmap R package [41] (version 1.10.1) with the regularized log-transformed counts from DESeq2 and row scaling turned on. Cell wall integrity pathway genes were taken from Rocha et al. [32] (Table S1). *A. flavus* orthologs of the *A. fumigatus* genes were identified using the reciprocal best-hit tool easy-rbh from MMseqs2 [42] (version 15.6f452) with a coverage cutoff of 0.5. To determine differences in gene expression of fungi treated with flavonoids, we evaluated the expression profiles of *A. flavus* genes that were putative to cell wall biosynthesis from *A. fumigatus* [43]. To determine differences in gene expression associated with GAG biosynthesis and signaling, *A. flavus* genes were identified using a reciprocal best hit with the *A. fumigatus* genes [44], and heatmaps were generated using RNA-seq data of flavonoid-treated fungi.

### 2.13. Statistical Analysis

Mycelial weight was measured by lyophilizing filtered mycelia for 24 h. Antioxidant assays of PDB media at 72 h (3 days) of incubation with AF3357 treated with flavonoids was measured by using the Trolox equivalent antioxidant capacity (TEAC), which was calculated as IC_50_ (Trolox)/IC_50_ (Sample). The IC_50_ values were calculated as recommended by the manufacturer (DPPH Antioxidant assay kit, Dojindo, Japan). To summarize, this process involved performing linear regression analysis and extrapolation of the concentration of Trolox or flavonoid to the 50% inhibition ratio percentage. Inhibition ratio percentage of flavonoid was calculated as follows: [Abs517 of blank1 (Buffer)−Abs517 of blank2 (Media buffer, ethanol, and assay buffer)]/[Abs517 of blank1 (Media buffer and assay buffer) × 100]. Inhibition ratio percentage of Trolox was calculated as follows: [(Abs517 of 0 µg/µL Trolox standard−Abs517 of blank3 (Ethanol and assay buffer))−(Abs517 of 40–80 µg/µL Trolox standard−Abs517 of blank3 (Ethanol and assay buffer))]/[(Abs517 of 0 µg/µL Trolox standard−Abs517 of blank3 (Ethanol and assay buffer))]. Graphical creation of mycelia and antioxidant assays was carried out using R (V4.1) [45] and Microsoft Excel. Normality was tested on measured variables, OD-560 nm in adhesion assays, ergosterol content, and single-gene expression qRT-PCR by using Shapiro’s test and the Q-Q plots of the residuals, if needed, and the data were normalized by performing logarithmic transformations. Gene expression, enzymatic assays, and cell adherence assays data were analyzed by performing one-way or two-way ANOVA by using the concentration of flavonoid and type of flavonoid as factors, followed by an honestly significant difference (HSD)–Tukey’s pairwise comparison test [46]. ANOVAs were performed for each time point separately. All data were analyzed with R (V4.1) [45].

## 3. Results

### 3.1. Flavonoid Effects on A. flavus While Grown on Corn-Enriched Plates

Bioassays using corn-enriched PDA were conducted to assess the impact of 0.001 µg/µL flavonoid treatments on AF3357 growth properties. The flavonoid treatment with 0.001 µg/µL of luteolin-7-glu resulted in a 10% reduction in maximum growth at day seven compared to DMSO and buffer (Figure S1 and Table 1). Although there were no differences in the maximum growth rate (µmax; Table 1), these results indicated that glycosylated luteolin slightly decreased the maximum growth of the fungi by 10%.

### 3.2. Flavonoids Damage the Surface of the A. flavus Cell Wall

**Figure 1:**
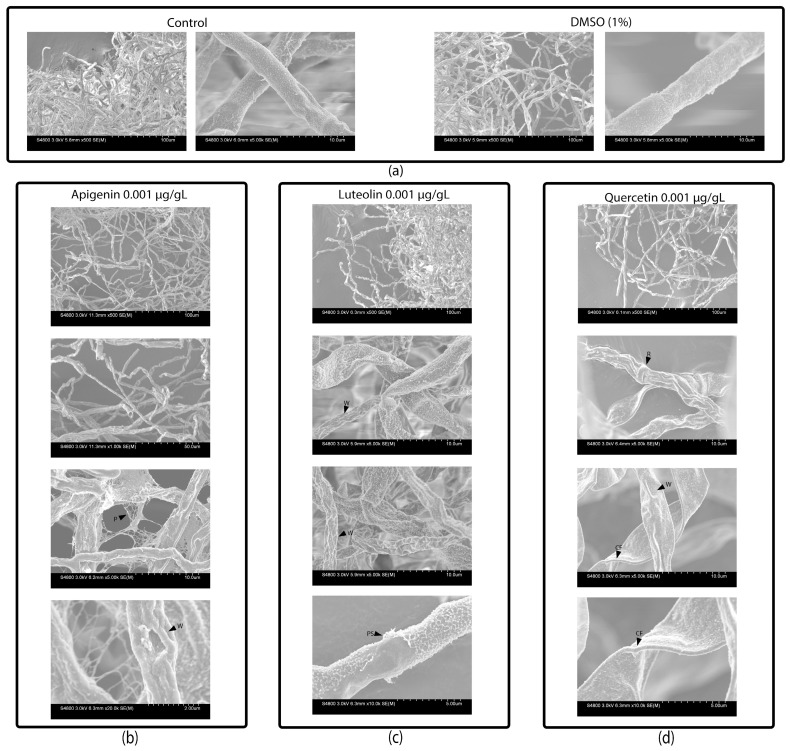
Effect of flavonoids on cell wall surface of *A. flavus* AF3357 after 72 h. Biological assays performed with AF3357 on PDB amended with (**a**) 0 µg/µL (1% DMSO and control without DMSO), (**b**) 0.001 µg/µL of apigenin, (**c**) 0.001 µg/µL of luteolin, and (**d**) 0.001 µg/µL of quercetin. White bars and ticks represent the scale of the microscope image. Subletters with black arrows indicate: ring-like structures (R), disintegration of mycelia (D), peeling on surface of mycelia (P), flattening/collapse of mycelia (F), and wrinkling of mycelial cell wall.

SEM imaging revealed differences in the physical structure of the mycelia treated with 0.001 µg/µL of flavonoids compared to the controls (Figure 1). Specifically, the apigenin, luteolin, and quercetin treatments induced the development of wrinkle-like structures on the surface of the mycelial fiber, leading to its collapse. The quercetin treatment resulted in extreme mycelial collapse, presenting as flat fibers. Apigenin and luteolin caused peeling of the outer layer of the mycelial cell wall, but apigenin also led to fiber detachment, creating a webbed-like mycelial cell wall formation. The luteolin treatments exhibited surface cell detachment that looked like vesicles and fibers.

### 3.3. Fungal Ergosterol Content Increased in Response to Flavonoids

**Figure 2:**
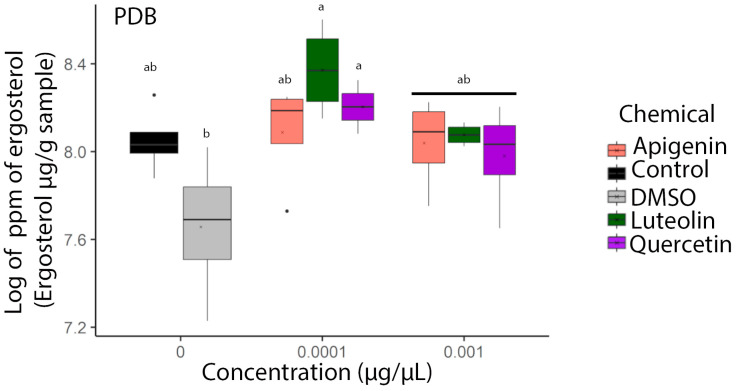
Flavonoids increased ergosterol content in *A. flavus* AF3357 at 72 h. Biological assays performed with AF3357 grown in PDB media amended with apigenin, luteolin, or quercetin at 0 (1% DMSO and control without DMSO), 0.0001, and 0.001 µg/µL concentrations. Color legend shows treatments used: salmon (apigenin), green (luteolin), purple (quercetin), black (control = 0% DMSO), and gray (1% DMSO). Different letters over box plots represent statistically significant differences according to HSD–Tukey’s assay (*p* < 0.05) for gene expression performed separately by day. Box-plot whiskers depict the maximum (25th—1.5 × interquartile range “IQR”) and minimum (75th percentile + 1.5 × interquartile range (IQR)), and the boxes depict median, first (25th percentile), and third (75th percentile) quantile distribution values (*n* = 3) per treatment.

**Figure 3:**
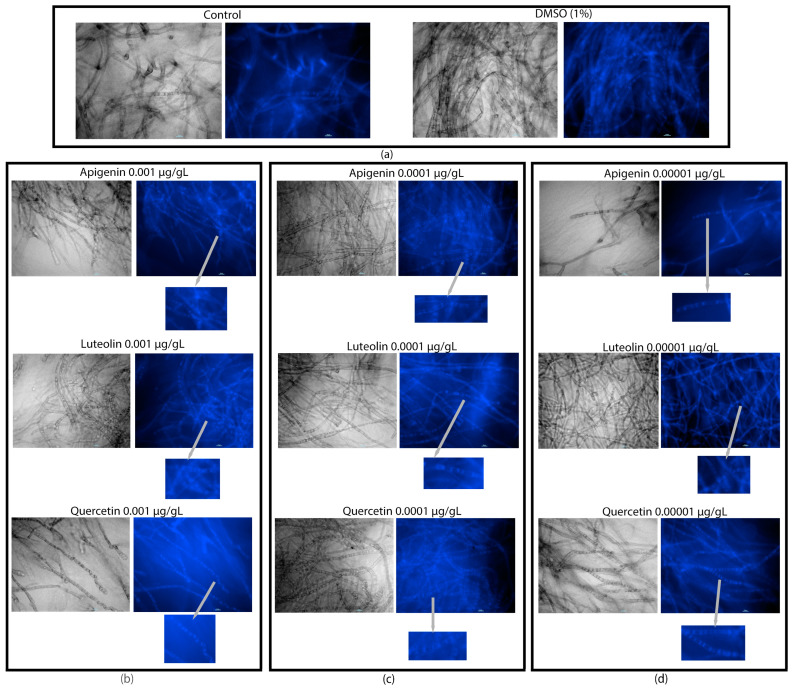
Effect of flavonoids on ergosterol localization in *A. flavus* AF3357 after 72 h incubation in PDB under shaking conditions. Biological assays performed with AF3357 exposed to apigenin, luteolin, and quercetin at (**a**) 0 (1% DMSO and control without DMSO), (**b**) 0.001, (**c**) 0.0001, and (**d**) 0.00001 µg/µL concentrations (*n* = 4) per treatment. Grey arrows point to a digital zoom of a section of the image. Green and blue bars represent the scale of magnification. The scale bars are all set to represent 10 µm.

We observed from the UPLC analysis that there was a significant increase in ergosterol content accumulation in the fungi grown in PDB and treated with 0.0001 µg/µL of luteolin and apigenin (Figure 2). Surprisingly, these differences in ergosterol content did not correlate with changes in the dry weight of the mycelia (Figure S2) or visible alterations in the mycelial appearance within the flask (Figure S3). Under both the control and flavonoid treatments, we noticed the formation of tight mycelial balls when the cultures were grown under shaking conditions (Figure S3). However, when growing under static conditions, the flavonoid-treated cultures exhibited small, well-defined mycelial balls, while the control cultures formed larger aggregates (Figure S4). To further investigate the localization of ergosterol, we used filipin staining. The resulting images revealed unique patterns, where ergosterol accumulation was concentrated at the edges of the cells and between cell boundaries, while a dark circular region devoid of ergosterol was observed in the middle of the cell (Figure 3).

### 3.4. Flavonoids Co-Localized in Vesicle-like Structures

**Figure 4:**
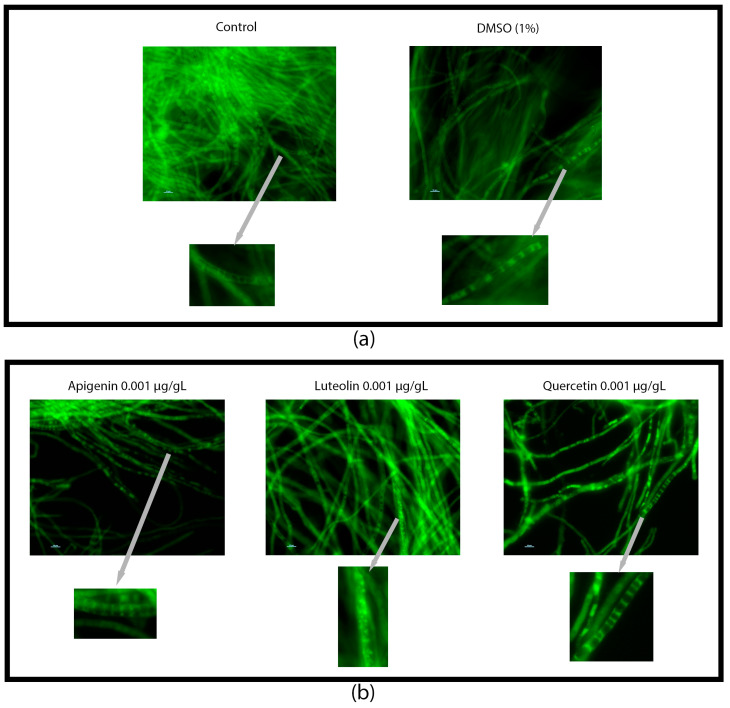
Flavonoids localize outside and inside fungal mycelia. DPBA assay visualized with fluorescence microscopy from AF3357 after 72 h incubation in PDB under static conditions. Biological assays performed with AF3357 exposed to apigenin, luteolin, and quercetin at (**a**) 0 (1% DMSO and control without DMSO) and (**b**) 0.001 µg/µL concentrations. Grey arrows point to a digital zoom of a section of the fluorescent image. White and blue bars represent the scale of magnification. The scale bars are all set to represent 10 µm.

We evaluated flavonoid localization on the mycelial surface by using DPBA assays of AF3357 incubated under shaking conditions. The fungal cells grown under shaking environments looked vacuolated and enlarged regardless of the treatment (Figure 4 and Figure S5). AF3357 grown under static conditions showed a similar vacuolation of the cells as in the shaking conditions. One major difference was observed under static conditions; the luteolin treatment showed fluorescent vesicle-like structures.

### 3.5. Flavonoid Effect on Osmotic Stress in AF3357

The results from our microscopy experiments showed physical changes and potential damage to the *A. flavus* cell wall surface after exposure to the apigenin, luteolin, and quercetin flavonoids. Certain compounds are known to function as osmotic stabilizers in the fungal cell wall, even in the presence of defects or a complete absence of the cell wall. To determine if flavonoid-induced cell wall defects were present, we exposed the flavonoid-treated mycelium to both an untreated medium and medium supplemented with osmotic stabilizers. We found that the *A. flavus* cultures grown on PDA medium showed no observable morphological defects when compared to the cultures grown in the presence of 0.6 M KCl or 1 M sorbitol A (Figure S6).

### 3.6. Fungal Cell Adherence Increased in Response to Flavonoids

**Figure 5:**
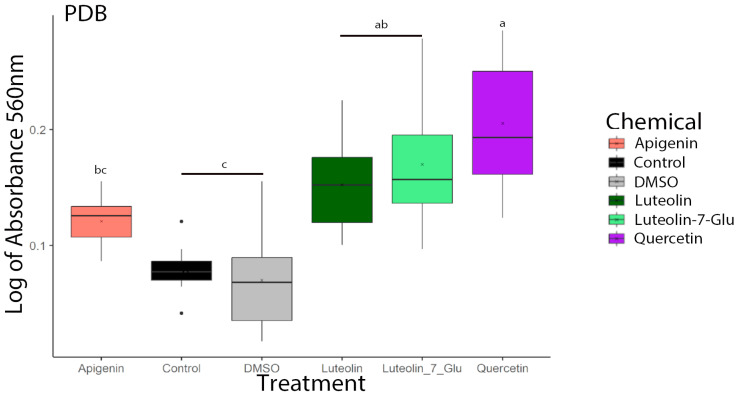
Flavonoids increased cell adhesion of *A. flavus* AF3357 at 72 h. Cell adhesion assay performed with AF3357 exposed to apigenin, luteolin, luteolin-7-glucoside, and quercetin at 0 (1% DMSO and control without DMSO) and 0.001 µg/µL concentrations. Color legend shows treatments used: salmon (apigenin), green (luteolin), mint (luteolin-7-glucoside), purple (quercetin), black (control = 0% DMSO), and gray (1% DMSO). Different letters over box plots represent significant differences according to HSD–Tukey’s assay (*p* < 0.05) for gene expression performed separately by day. Box-plot whiskers depict the maximum (25th—1.5 × interquartile range “IQR”) and minimum (75th percentile + 1.5 × interquartile range (IQR)), and the boxes depict the median, first (25th percentile), and third (75th percentile) quantile distribution values (*n* = 8) per treatment.

To quantify the capacity of the fungal biofilms to adhere to a surface, we performed an adhesion bioassay. The adhesion assay showed that AF3357 treated with 0.001 µg/µL of luteolin, luteolin-7-Glu, and quercetin had higher adhesion properties compared to the control and DMSO treatments by promoting cell adherence and biofilm formation on the fungus cell surface (Figure 5).

### 3.7. Flavonoids Have Different IC_50_ Values and Antioxidant Capacities

To assess the impact of flavonoid-treated AF3357 on medium oxidative levels, we conducted an antioxidant capacity assay using filtered broth after 72 h of incubation. The IC_50_ values from the filtered broth revealed that the quercetin-treated fungi exhibited the highest antioxidant value (0.0187), followed by apigenin (0.0162) and then luteolin (0.01) (Table 2). The IC_50_ value represents the flavonoid concentration required to reach 50% of the total antioxidant capacity in the media. Notably, luteolin facilitated IC_50_ inhibition at lower concentrations compared to the other flavonoids. Additionally, luteolin had the highest Trolox equivalent antioxidant capacity (TEAC) compared to the other compounds, indicating that AF3357 treated with luteolin exhibited superior antioxidant capacity in the environment compared to apigenin and quercetin.

### 3.8. Flavonoids Increased Expression of Genes Related to Putative Development and Cell Wall Biosynthesis

Due to the differences in ergosterol measured (Figure 2), we investigated the impact of flavonoids on genes associated with the fungal cell wall biosynthetic pathway (Table S1). Notably, glucan synthase (*AFLA_052800*), responsible for polysaccharide biosynthesis and proper cell wall assembly, exhibited significantly higher expression under the luteolin and apigenin treatments (0.0001 µg/µL) at 16 (luteolin and apigenin) and 24 h (luteolin) (Figure S7) compared to DMSO. Chitin synthase (*AFLA_136030*), involved in the formation of chitin deposition, showed significant higher changes in gene expression at 16 h (0.0001 µg/µL) and displayed a significantly lower expression when treated with quercetin (0.001 µg/µL) after 24 h compared to the control DMSO (Figure S7). Additionally, cell wall mannoprotein 1 (*AFLA_039410*), which contributes to cell wall rigidity and shape, showed significantly higher expression with the quercetin treatment (0.0001 µg/µL) at 24 h compared to the control DMSO and at 72 h when compared to the control (Figure S7).

To explore if flavonoids affect fungal developmental programs, we assessed the expression of genes related to the regulation of morphological development. A gene involved in intracellular signal transduction, conidiophore biogenesis, and sterigmatocystin production in *A. nidulans*, *flbA*, showed no changes in expression at 16 h (Figure S8). However, at 24 h, *flbA* showed significantly higher expression when treated with 0.0001 and 0.001 µg/µL of apigenin, luteolin, and quercetin compared to the controls (Figure S8). Expression of *sclR*, a gene involved in the positive regulation of filamentous growth and development, also increased under the treatments of 0.001 µg/µL of apigenin and luteolin compared with the DMSO treatment at 24 h and with 0.001 µg/µL of apigenin, luteolin, and quercetin compared with the control at 72 h (Figure S8). Because there were no significant differences in *sclR* expression compared with the control (0% DMSO), there was a potential interaction effect between DMSO and the flavonoids.

### 3.9. Fungal Gene Expression Changes in Response to Flavonoids

**Figure 6:**
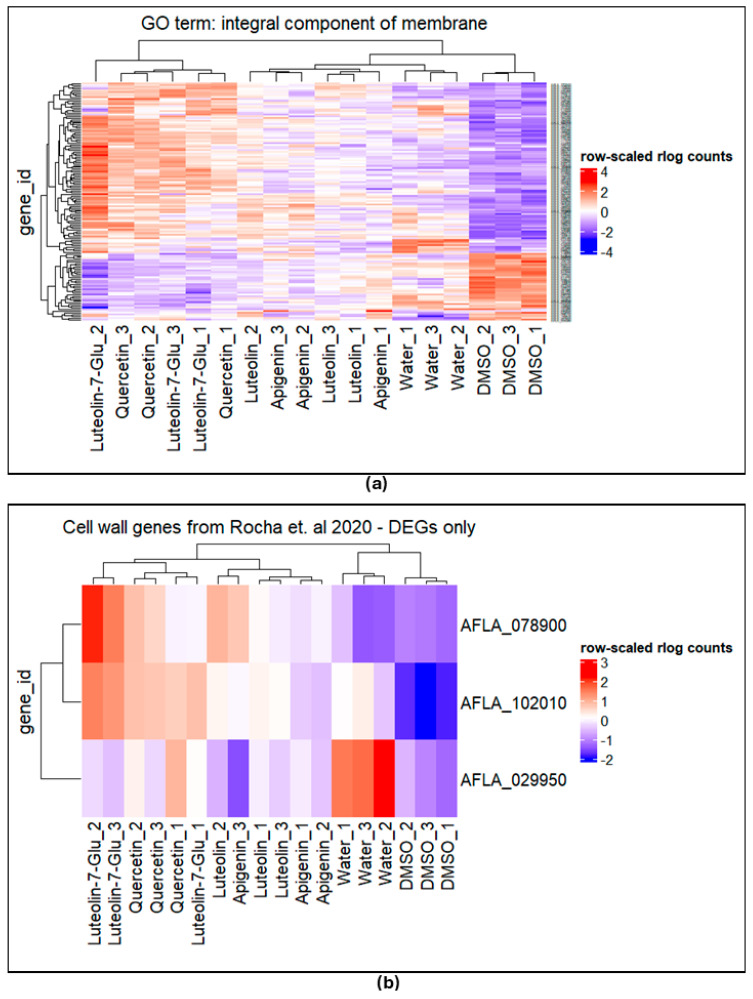
Hierarchical clustering of gene expression. AF3357 treated with 0.001 µg/µL of flavonoids: apigenin, luteolin, luteolin-7-glucoside (Lut_7_Glu), and quercetin, 1% DMSO, or buffer control. (**a**) Genes significantly enriched in the integral component of membrane and (**b**) genes significantly enriched in cell wall and in Rocha et al., 2020 [43].

Due to the differences in ergosterol content (Figure 2) and the targeted gene expression results of cell wall assembly and composition (measured with glucan synthase *AFLA_052800*, chitin synthase *AFLA_136030*, and cell wall mannoprotein 1 *AFLA_039410*) (Figure S8), we conducted an RNA-seq analysis on AF3357 treated with 0.001 µg/µL of flavonoids (apigenin, luteolin, luteolin-7-glucoside, and quercetin), 1% DMSO, or buffer control for 72 h. The analysis revealed that genes from the flavonoid-treated cultures tended to cluster together (Figure 6). Compared to DMSO, the flavonoids significantly increased the expression of 178-genes (luteolin), 396-genes (quercetin), 502-genes (luteolin-7-glucoside), and 159-genes (apigenin), while decreasing the expression of 31-genes (luteolin), 142-genes (quercetin), 166-genes (luteolin-7-glucoside), and 26-genes (apigenin) (Table 3).

Gene ontology enrichment analysis of the significantly differentially expressed genes showed an enrichment in extracellular processes, integral components of membrane, and transmembrane transport (Table 4). Hierarchical clustering analysis of genes associated with the integral components of the membrane indicated that the flavonoid treatments tended to upregulate the expression of genes in this category compared to DMSO and water (Figure 6a). Similarly, homologs of putative cell wall biosynthesis genes [43] exhibited significantly higher expression when treated with flavonoids compared to DMSO and buffer (Figure 6b). However, putative GAG biosynthesis and GAG signaling showed no significant differences in expression when treated with flavonoids compared to DMSO and the buffer control (Figure S7).

## 4. Discussion

In this study, we found that luteolin and quercetin increased the biosynthesis of ergosterol, leading to an increased localization of this molecule at the edges of fungal cells. Ergosterol is a crucial membrane sterol essential for fungal growth, development, and regulation of membrane fluidity [47]. It also serves as a target for antifungal drug development in human infections [48]. Interestingly, ergosterol biosynthesis in yeast is linked to resistance to air-drying, making it vital for fungal survival during environmental dry/wet cycles [49]. In human research focused on *A. fumigatus* infections, triazoles are commonly used to inhibit ergosterol biosynthesis, effectively depleting the fungi [48]. Amphotericin B (AMB) is a different type of antifungal drug for use in humans that induces cell death by acting as an oxidizing agent, allowing hydrogen peroxide to permeate through the fungal membranes and cause lipid peroxidation [50]. Ergosterol plays a role in managing oxidative stress [51], helping to fortify the cell wall against free radicals that lead to harmful oxidation in the cell [52]. Research on *Aspergillus terreus* resistance to AMB showed that this drug does not alter ergosterol levels but instead promotes the production of catalases in the fungus [50]. Catalases protect against oxidative damage of fungal cell membranes [50], which could be a compounded effect, in addition to flavonoid treatments, resulting in differences in antioxidant capacity in fungi treated with these compounds. In response to oxidative stress damage to the surface of the cell wall, fungal protective mechanisms involve synthesizing additional cell wall components and increasing the production of exopolysaccharides to thicken the cell wall [53,54]. Although the molecular mechanism linked to the production of cell wall components in response to oxidative stress is not fully elucidated, these protective responses could be linked to the observed increase in ergosterol production in AF3357 treated with luteolin and quercetin.

We found that flavonoids influenced the environment in which AF3357 grows by leading to different levels of antioxidant capacity in the media (Table 1). We detected that quercetin had the lowest antioxidant capacity among the flavonoids tested, leading to higher oxidative stress levels in the media compared to apigenin and luteolin. In general, flavonoids are compounds that mitigate oxidative damage under abiotic stress conditions in fungi [55]. Our results imply that flavonoids may have an effect on AF3357 that can lead to differences in the antioxidant capacity of the environment and that they are associated with physiological and molecular changes in the fungus. Lower antioxidant capacity can be indicative of certain oxidative-stress-originated events [52] in the fungus. Most of the research evaluating the effects of flavonoids on corn plant–microbe interactions involve soil microbiome studies, although the relationships between flavonoids and microbiomes in other plant tissues such as silk and seeds are less understood. For example, in corn cobs, silks have high levels of flavonoids, polysaccharides, steroids, polyphenols, and other substances [56,57]. These high levels of flavonoids in silks have been linked to insect-resistant traits [57]. In other tissues like corn kernels, higher levels of flavonoids (anthocyanins and phlobaphenes) lead to higher antioxidant capacity [58], which could be associated with agronomically important traits such as fungal growth and mycotoxin contamination resistance in corn. In fungi, changes in the redox environment surrounding the cell (either positive or negative deviations from normality) have been shown to activate signaling cascades involved in regulating apoptosis and programmed cell death [59]. This is likely what caused the observed physical damage to the surface of the AF3357 cells treated with the flavonoids.

We observed vesicle formation in the fungal cells treated with luteolin. These vesicles colocalized with luteolin and may play a role in the cellular import/export mechanism. In plants, flavonoids are transported to vacuoles for storage or to other compartments, including extracellular space [16]. In plants, there are three possible modes of transport for flavonoids: (1) vesicle trafficking, (2) membrane transporters, and (3) glutathione-S-transferase (GST)-mediated transport [16,60]. Notably, there is limited information about fungal methods of transport for flavonoids, perhaps because it is assumed that these molecules tend to be produced by plants. There is evidence that *Stropharia rugosoannulata* (an edible mushroom) produces flavonoids [61] and that species of *Aspergillus* such as *Aspergillus candidus* are able to produce chlorflavonin, an antibiotic flavonoid [62,63]. Also, analysis has shown that fungal strains have the capacity to degrade flavonoids in *A. niger* 1344 [64]. If flavonoids can be produced and degraded by fungi, there might be a transport method that involves vesicles. For example, in *A. parasiticus* and *A. flavus,* aflatoxin production occurs in specialized vesicles that are called “aflatoxisomes” [65,66]. These aflatoxisomes carry the aflatoxin compounds to the plasma membrane, where they fuse with the plasma membrane and export the aflatoxin to the extracellular environment via exocytosis [65,66]. We hypothesize that *A. flavus* might transport flavonoids that are either imported from the environment or potentially biosynthesized by fungi in a similar method employed by aflatoxisome. Further research is needed to discern the intra- and extracellular transportation methods of flavonoids in *Aspergillus* and in fungi in general.

Due to the microscopic changes observed on the surface of the *A. flavus* cell wall upon exposure to flavonoids, we hypothesized that flavonoids lead to internal cell wall structural defects in *A. flavus*. To test this hypothesis, we grew *A. flavus* under flavonoid exposed and non-exposed conditions prior to transferring the mycelium to media with and without supplemented osmotic stabilizers. We chose this method because studies utilizing mutant strains of *Saccharomyces cerevisiae* and *A. nidulans* have demonstrated that osmotic stabilizers, which can reduce cellular turgor pressure, can be used to restore wild-type growth in the presence of cell wall defects [67,68,69]. If the flavonoid-exposed mycelium contained any potential cell wall defects, we expected to see defective growth on only the PDA plates and normal wild-type growth on the plates supplemented with osmotic stabilizers. Our results showed no observational morphological defects in the cultures exposed to the flavonoids and in those grown on only PDA when compared to the controls. Despite observing no morphological defects in the flavonoid-exposed PDA cultures, it is still possible that cell wall defects may have been present. There is evidence that flavonoids act as antifungal agents; for example, calchone inhibits *A. fumigatus* at a minimum inhibitory concentration (MIC) of 4–16 µg/mL, flavones like apigenin have an of MIC of 5 µg/mL, luteolin-7-O-β-D-glucuronide leads to inhibition of *A. niger* with an MIC of 7.5–62.5 µg/mL, luteolin inhibits *A. fumigatus* with an MIC of 3.9–83 µg/mL, and naringenin leads to inhibition of *A. flavus* with an MIC of 3.125 mg/mL [12,15,70]. The overall mechanisms of inhibition of these compounds are associated with the inhibition of ion efflux pumps, the disruption of cell division, affecting RNA/DNA/protein biosynthesis, the disruption of plasma membranes, mitochondrial dysfunction, and interfering with cell wall formation [12,15,70]. It is possible that cell wall defects were remediated after transferring the flavonoid-exposed mycelium to PDA medium and allowing the mycelium to grow for 72 h. Future experiments such as chemical cell wall analyses will be conducted to study the *A. flavus* cell wall composition in greater detail upon exposure to flavonoids.

In our experiments, we observed tight mycelia aggregates when AF3357 was grown under shaking conditions for both the control and flavonoid treatments. However, when the cultures were grown under static conditions, mycelia aggregates were more pronounced in the flavonoid-treated samples compared to the DMSO controls. Specifically, luteolin and quercetin exposure enhanced the ability of *A. flavus* to adhere to a surface, suggesting a potential role in modulating fungal aggregation and community dynamics. It is well known that adherence is a precursor to biofilm formation [71]. In rice, CRISPR plant mutants with increased apigenin content in their roots exhibited biofilm formation in soil diazotrophs under nitrogen-limited and aerobic conditions [18]. This protective biofilm promoted bacterial nitrogen fixation and enhanced root colonization [18]. Flavonols, likely due to their membrane-lytic properties, led to bacterial aggregation, but this effect did not necessarily support biofilm production [24]. We found higher levels of cell adhesion in the AF3357 treated with flavonoids. These findings are contrary to previous biofilm studies [24] focused on *Staphylococcus aureus* [72] and *Streptococcus mutans* [73], and these results could be explained by the differences in biological systems because our results are specific to *A. flavus*. These differences in adhesion properties can be explained by the biphasic effect of flavonoids as antioxidants and as promoters of oxidative stress [74]. Fungi must be able to quicky adapt to stressors encountered in the environment, where in this case, the flavonoids are acting as a “stressor” because they are changing the redox environment around the fungal cell by scavenging reactive oxygen species [75].

Ample research has been carried out in bacterial biofilm formation linked to plant diseases [76,77,78,79], although less research has been carried out in this area in plant pathogenic fungi. It has been reported that fungi linked to plant diseases have biofilm-like properties [79,80] that could aid in defense against other organisms and environmental stress [79,80,81], and in some cases, biofilm formation exhibited by beneficial microorganisms can lead to land rehabilitation [82]. Biofilms consist of an extracellular matrix (ECM) containing extracellular DNA, polyols, proteins, lipids, and exopolysaccharides (EPS) such as α-glucans, galactomannan, and galactosaminogalactan (GAG) [44]. Genes involved in ECM formation include *uge3* (glucose 4-epimerase), *gtb3* (a putative transmembrane glycosyltransferase), *ega3*, *sph3* (glycoside hydrolases), and *agd3* (a secreted polysaccharide deacetylase that mediates deacetylation of GalNAc residues within GAG). Additionally, *agsB* is implicated in forming glucan chains, although the precise role of α-glucans in biofilm formation remains unknown [44]. In *A. fumigatus,* regulation of biofilm formation involves several transcription factors genes, including f*lo8*, *ada2*, and e*fg1* [44]. However, when we evaluated the expression of putative signaling genes in *A. flavus*, from these *A. fumigatus* transcription factors [44], we found no differences in gene expression levels in the flavonoid-treated samples when compared to the control samples. GAG has been shown to play a role in virulence in *A. fumigatus* [83], and although we did not detect changes in the expression of putative GAG biosynthesis genes in *A. flavus,* it is possible that these biosynthesis pathways may only be differentially expressed in planta or while infecting viable corn kernels. Furthermore, the genetic regulatory pathways governing GAG biosynthesis are well documented in some fungal species but are not as well understood in *A. flavus*. We evaluated the expression of known GAG biosynthesis genes based on orthologous genes that have been functionally characterized in other fungal species to play a role in GAG production. It is possible that we did not detect a difference in the expression of putative GAG biosynthesis genes in *A. flavus* due to the fact that the genetic regulatory pathways governing the production of this compound may be different in *A. flavus* from those of other species.

In summary, we determined that the flavonoid treatments on AF3357 led to differences in oxidative stress levels in the environment, which co-occurred with changes in cell wall structure, increased the ability of the fungus to adhere to a surface, and caused physical damage to the outer layer of the fungal cell wall. Additionally, the flavonoid treatments led to increases in ergosterol content coupled with increases in the expression of genes that are thought to regulate asexual development [84]. It is possible that the gene expression of specific developmental genes was altered during exposure to the flavonoid treatments in a similar way as the ergosterol content. However, the molecular link between the changes in ergosterol content and asexual development in *A. flavus* under flavonoid treatments remains unknown. Additionally, other cell wall components that contribute to cell wall integrity and rigidity may be unable to prevent cell collapse, although these phenotypes do not lead to osmotic stress changes. What these components are is yet to be determined, in addition the role of the putative cell wall and membrane genes in *A. flavus*. The molecular link between flavonoids and oxidative stress, cell wall phenotypic changes, increases in gene expression of filamentous growth, and increases in cell adhesion and biofilm formation in *A. flavus* are not known. Fungal biofilm formation could be a target for controlling plant disease management that can lead to a lower use of fungicide applications [79], although further research is needed to understand the effects of pathogenic fungi biofilm formation in agriculture management. Perhaps the formation of vesicles in the luteolin treatment indicate that the flavonoids are taken into the cell, where they might act as messaging molecules that alter physiology and gene expression in fungi.

## Figures and Tables

**Table 1:** Effect of flavonoids on modeled growth curves of *A. flavus* AF3357. Mycelium was treated with 0.001 µg/µL of flavonoids or controls (DMSO or buffer). Y_0_ is initial value of growth/abundance, µmax is the maximum growth rate (1/time), K is the maximum growth/abundance (carrying capacity), H_0_ is the lag phase (max growth rate × lag phase), and r^2^ is the regression error metric.

Treatment	Y_0_	Mumax	K	h_0_	R^2^
Apigenin	0.1	1.54	7.58	1.73 × 10^−7^	0.95
Buffer	0.1	1.57	7.65	2.67 × 10^−7^	0.98
DMSO	0.1	1.54	7.53	3.50 × 10^−7^	0.97
Luteolin	0.1	1.53	7.13	4.93 × 10^−7^	0.92
Luteolin_7_Glu	0.1	1.58	6.71	3.04 × 10^−7^	0.92
Quercetin	0.1	1.62	7.02	2.36 × 10^−7^	0.88

**Table 2:** Antioxidant capacity assay performed in *A. flavus* AF3357 after three days of incubation with flavonoids in PDB media.

Treatment	IC_50_ *	TEAC IC_50_ (Trolox)/IC_50_ (Sample)
Apigenin	0.0162	4.0196
Luteolin	0.0100	6.5045
Quercetin	0.0187	3.4832
Trolox	0.0652	-

* IC_50_ is the concentration of compound where 50% of the antioxidant capacity is achieved.

**Table 3:** Gene expression comparisons from AF3357 treated with 0.001 µg/µL of flavonoids (apigenin, luteolin, luteolin-7-glucoside, and quercetin), 1% DMSO, or buffer control for 72 h.

Comparison	Upregulated	Downregulated
Luteolin_vs_DMSO	178	31
Quercetin_vs_DMSO	395	142
Luteolin_7_Glucoside_vs_DMSO	502	166
Apigenin_vs_DMSO	159	26
DMSO_vs_Water	73	207

**Table 4:** Gene expression enrichment analysis of RNA-seq from AF3357 treated with 0.001 µg/µL of flavonoids (apigenin, luteolin, luteolin-7-glucoside, and quercetin), 1% DMSO, or buffer control.

Comparison	Direction	TermID	*p* Value Adjusted
DMSO_vs_Buffer	down	Extracellular	1.36351 × 10^−7^
Luteolin_vs_DMSO	up	Extracellular	5.02456 × 10^−6^
DMSO_vs_Water	down	SMURF_cluster_52	0.000115558
Lut_7_Glu_vs_DMSO	up	Extracellular	0.000247728
Luteolin_vs_DMSO	down	Integral component of membrane	0.000685218
Quercetin_vs_DMSO	up	Extracellular	0.001372928
Quercetin_vs_DMSO	up	Valine, leucine and isoleucine biosynthesis	0.0040794
Lut_7_Glu_vs_DMSO	up	Integral component of membrane	0.004143167
Lut_7_Glu_vs_DMSO	up	Transmembrane transport	0.004143167
Quercetin_vs_DMSO	up	Pantothenate and CoA biosynthesis	0.007470882
Luteolin_vs_DMSO	up	Valine, leucine, and isoleucine biosynthesis	0.009795063
Luteolin_vs_DMSO	up	Pantothenate and CoA biosynthesis	0.012355729
Lut_7_Glu_vs_DMSO	up	Valine, leucine, and isoleucine biosynthesis	0.019079722
Apigenin_vs_DMSO	up	Lysosome/Vacuole	0.023625989
Apigenin_vs_DMSO	up	Extracellular	0.023625989
Lut_7_Glu_vs_DMSO	up	Pantothenate and CoA biosynthesis	0.033564725

## Data Availability

The raw sequencing reads can be accessed through NCBI bioproject number PRJNA1148494.

## Supplementary Materials

The following supporting information can be downloaded at: https://www.mdpi.com/article/10.3390/jof10090665/s1, Figure S1. Effect of flavonoids on growth of *A. flavus* 3357. AF3357 was grown on corn-enriched PDA treated with 0.001 µg/µL of flavonoids or controls (DMSO or buffer). Figure S2. Effect of flavonoids on mycelia weight of *A. flavus* 3357 at 72 h. Biological assays performed with AF3357 strain grown in PDB media that was exposed to apigenin, luteolin, and quercetin at 0 (1% DMSO and control without DMSO), 0.0001, and 0.001 µg/µL concentrations. Key color legend represents chemicals used: salmon: apigenin, green: luteolin, purple: quercetin, black: control (0% DMSO), and gray: DMSO (1% DMSO). Different letters over box plots represent statistically significant differences according to HSD–Tukey’s assay (*p* < 0.05) for gene expression performed separately by day. Box-plot whiskers depict the maximum (25th—1.5 * interquartile range “IQR”) and minimum (75th percentile + 1.5 * interquartile range (IQR)), and the box depicts median, first (25th percentile), and third (75th percentile) quantile distribution. *n* = 3 per treatment. Figure S3. Setup of the bioassay of *A. flavus* 3357 after 72 h incubation in PDB under shaking conditions. Biological assays performed with AF3357 strain exposed to apigenin, luteolin, and quercetin at (A) 0 (1% DMSO and control without DMSO), (B) 0.001, (C) 0.0001, and (D) 0.00001 µg/µL concentrations. *n* = 3 or 4 per treatment. Figure S4. Setup of the bioassay of *A. flavus* 3357 after 72 h incubation in PDB under static conditions. Biological assays performed with AF3357 strain exposed to apigenin, luteolin, and quercetin at (A) 0 (1% DMSO and control without DMSO) and (B) 0.001 µg/µL concentrations. *n* = 4 per treatment. Figure S5. Flavonoids localized outside and inside fungal mycelia. DPBA assay visualized with fluorescence microscopy from AF3357 after 72 h incubation in PDB under shaking conditions. Biological assays performed with AF3357 strain exposed to apigenin, luteolin, and quercetin at (A) 0 (1% DMSO and control without DMSO) and (B) 0.001 µg/µL concentrations. White and blue bars represent the scale of magnification. The scale bars are all set to represent 10 µm. Figure S6. Exposure to osmotic stabilizers did not reveal obvious flavonoid-induced cell wall defects in *A. flavus* mycelium. In order to determine if the flavonoid-treated *A. flavus* mycelium harbored cell wall defects, the AF3357 strain was first grown in the presence and absence of various flavonoid treatments prior to shifting the mycelium on PDA medium and PDA medium supplemented with 0.6 M KCl and 1 M sorbitol. The cultures were allowed to incubate for 72 h at 31 °C prior to observing the cultures for obvious morphological defects and photographing, and the experiment was carried out in triplicate. Figure S7. Effect of flavonoids on gene expression of cell wall biosynthesis-related genes from *A. flavus* 3357 at 16, 24, and 72 h. Gene expression of (A) *AFLA_052800* (glucan synthase component), (B) *AFLA_136030* (chitin synthase), and (C) *AFLA_039410* (cell wall mannoprotein 1) genes in biological assays performed with AF3357 strain exposed to apigenin, luteolin, and quercetin at 0 (1% DMSO and control without DMSO), 0.0001, and 0.001 µg/µL concentrations. Key color legend represents chemicals used: salmon: apigenin, green: luteolin, purple: quercetin, black: control (0% DMSO), and gray: DMSO (1% DMSO). Different letters over box plots represent statistically significant differences according to HSD–Tukey’s assay (*p* < 0.05) for gene expression performed separately by day. Box-plot whiskers depict the maximum (25th—1.5 * interquartile range “IQR”) and minimum (75th percentile + 1.5 * interquartile range (IQR)), and the box depicts median, first (25th percentile), and third (75th percentile) quantile distribution. *n* = 3 per treatment. Figure S8. Effect of flavonoids on expression of fungal-development-related genes from *A. flavus* 3357 at 16 and 24 h. Gene expression of (A) *flbA* and (B) *sclR* genes in biological assays performed with AF3357 strain exposed to apigenin, luteolin, and quercetin at 0 (1% DMSO and control without DMSO), 0.0001, and 0.001 µg/µL concentrations. Key color legend represents chemicals used;:salmon: apigenin, green: luteolin, purple: quercetin, black: control (0% DMSO), and gray: DMSO (1% DMSO). Different letters over box plots represent statistically significant differences according to HSD–Tukey’s assay (*p* < 0.05) for gene expression performed separately by day. Box-plot whiskers depict the maximum (25th—1.5 * interquartile range “IQR”) and minimum (75th percentile + 1.5 * interquartile range (IQR)), and the box depicts median, first (25th percentile), and third (75th percentile) quantile distribution. *n* = 3 per treatment. Figure S9. Sample variation in RNA-seq experiment. Principal component analysis of gene expression sequencing data from AF3357 treated with 0.001 µg/µL of flavonoids (apigenin, luteolin, luteolin-7-glucoside, and quercetin), 1% DMSO, or buffer control. Figure S10. Effect of flavonoids on GAG biosynthesis and signaling pathways. Hierarchical heatmap gene expression from AF3357 treated with 0.001 µg/µL of flavonoids (apigenin, luteolin, luteolin-7-glucoside (Lut_7-Glu), and quercetin), 1% DMSO, or buffer control. Putative genes in *A. flavus* that are in the A) GAG biosynthesis pathway and B) GAG signaling pathways. Table S1. Genes used for expression analysis in flavonoid bioassays.
