# Supplementary material for: Investigating the Impact of Flavonoids on Aspergillus flavus: Insights into Cell Wall Damage and Biofilms
Source: J Fungi (Basel). 2024 Sep 23;10(9):665. doi: 10.3390/jof10090665 (PMC11433479; doi:10.3390/jof10090665)
Supplement: Supplementary file 1 [file jof-10-00665-s001.zip › JoF-Supplemental_Materials_August_29_2024.pdf]

Figures and Tables

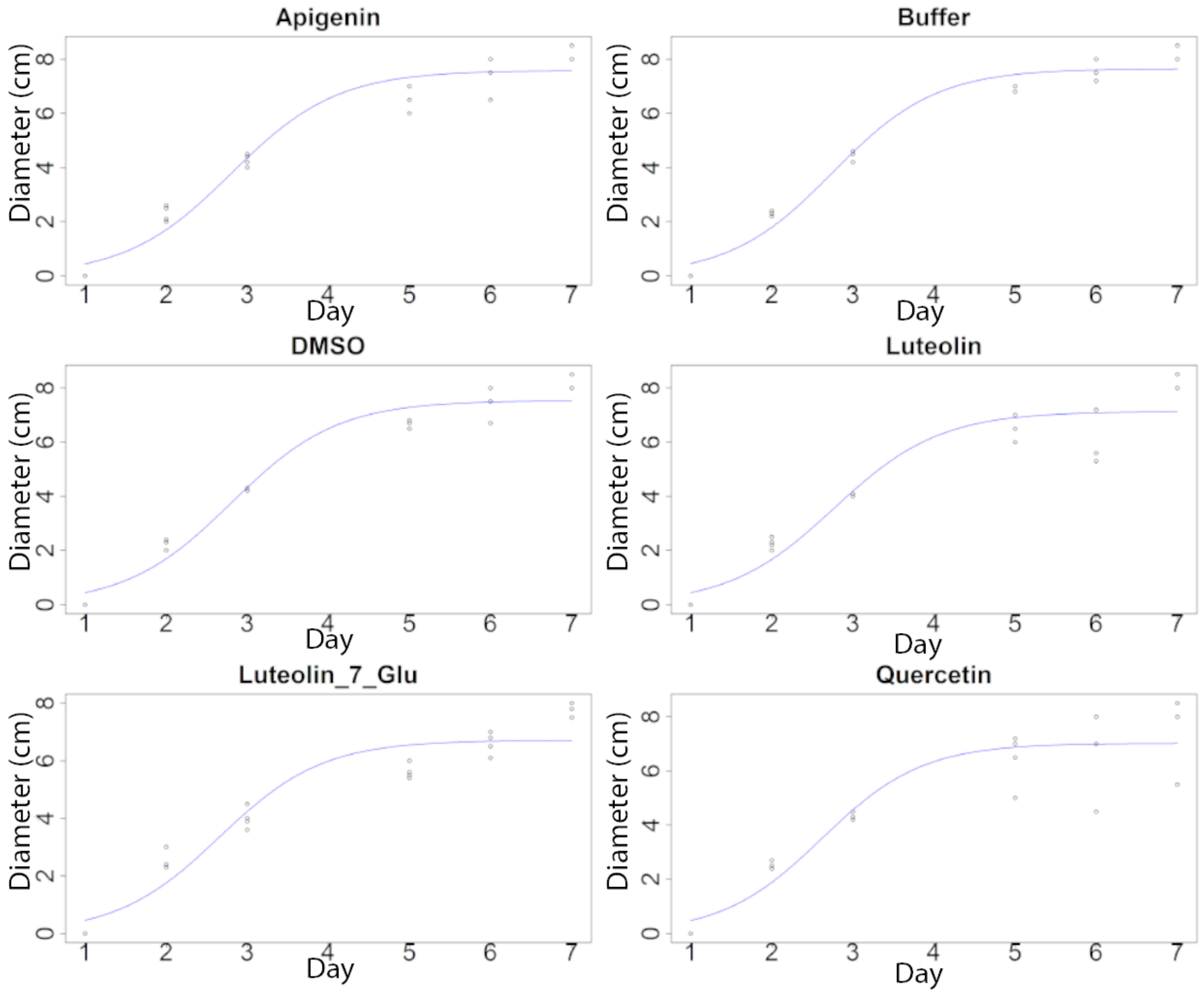

Figure S1. Effect of flavonoids on growth of *A. flavus* 3357. AF3357 was grown on corn enriched PDA treated with 0.001 µg/µL of flavonoids or controls (DMSO or buffer).

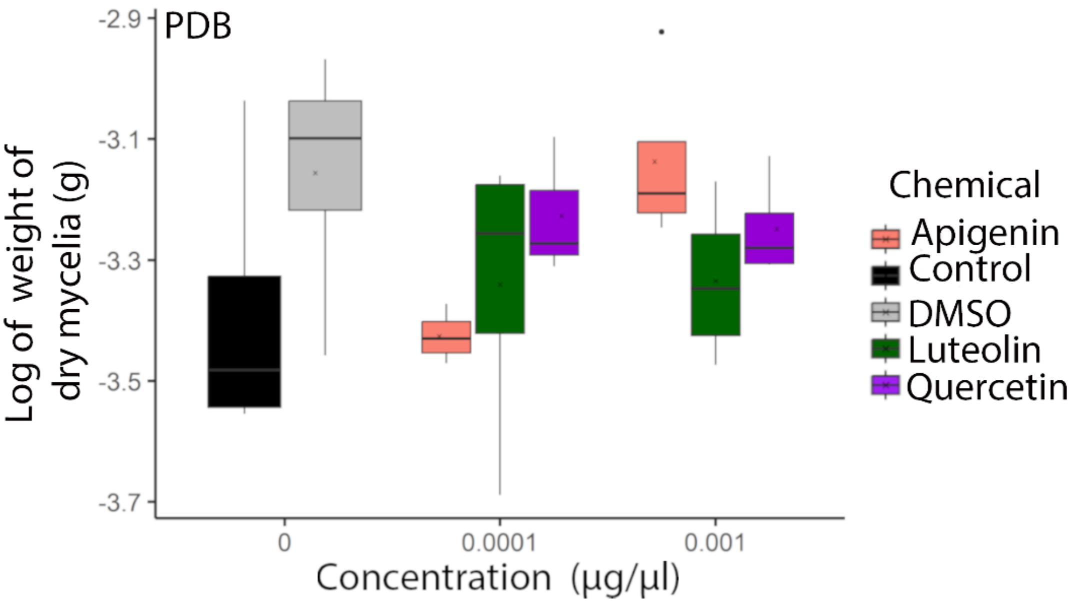

Figure S2. Effect of flavonoids on mycelia weight of *A. flavus* 3357 at 72 h. Biological assays performed with AF3357 strain grown in PDB media that was exposed to apigenin, luteolin, and quercetin at 0 (1% DMSO and control no-DMSO), 0.0001, and 0.001 µg/µL concentrations. Key color legend represents chemicals used; salmon: apigenin, green: luteolin, purple: quercetin, black: control (0% DMSO) and gray: DMSO (1% DMSO). Different letters over box plots represent statistically significant differences according to Tukey HSD assay ($p < 0.05$) for gene expression performed separately by day. Box-plot whiskers depict the maximum (25th - 1.5 * interquartile range "IQR") and minimum (75th percentile + 1.5 * interquartile range (IQR)), and the box depicts median,

first (25th percentile) and third (75th percentile) quantiles distribution. N = 3 per treatment.

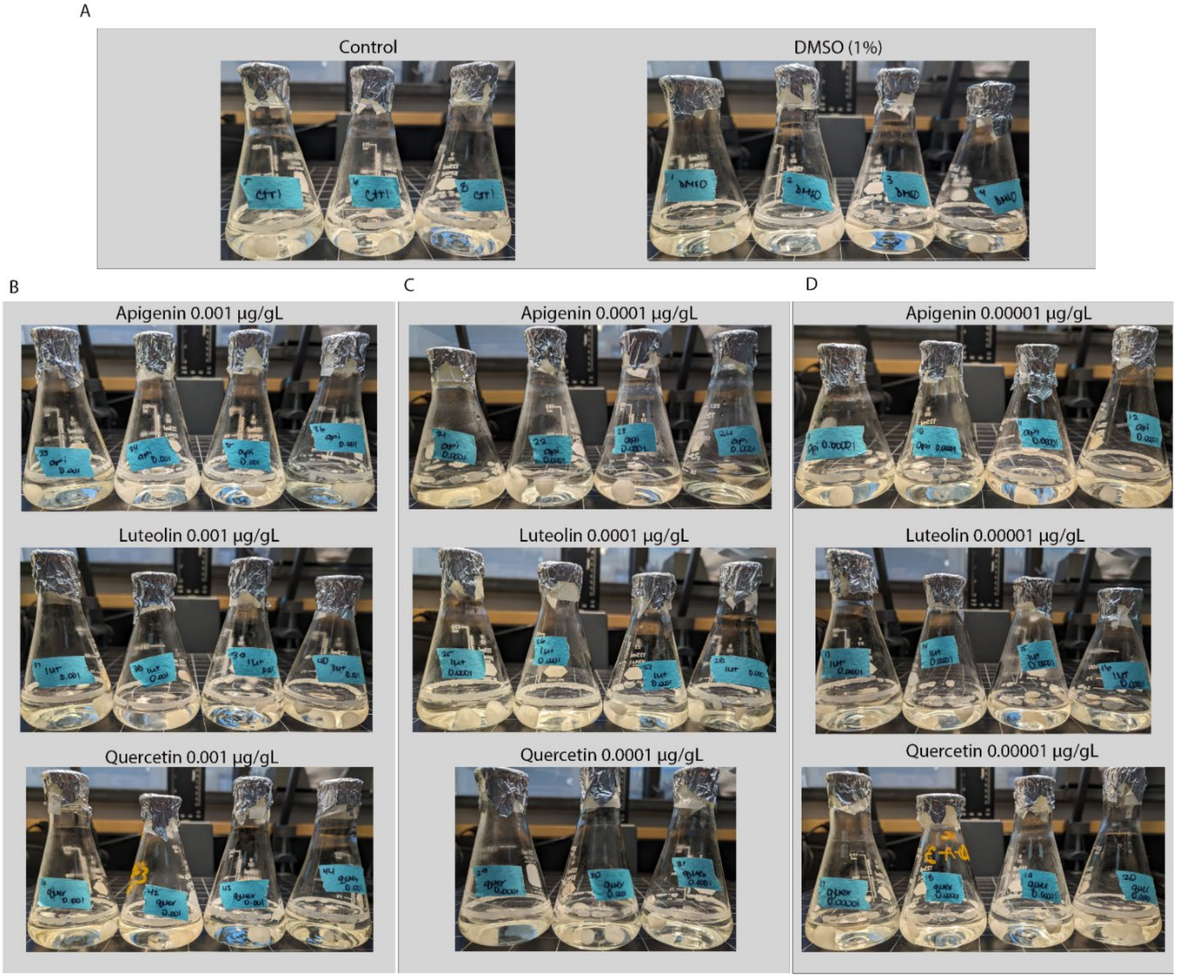

Figure S3. Set up of the bioassay of A. flavus 3357 after 72 h incubation in PDB under shaking conditions. Biological assays performed with AF3357 strain exposed to apigenin, luteolin, and quercetin at A) 0 (1% DMSO and control no-DMSO), B) 0.001, C) 0.0001, and D) 0.00001 µg/µL concentrations. N = 3 or 4 per treatment.

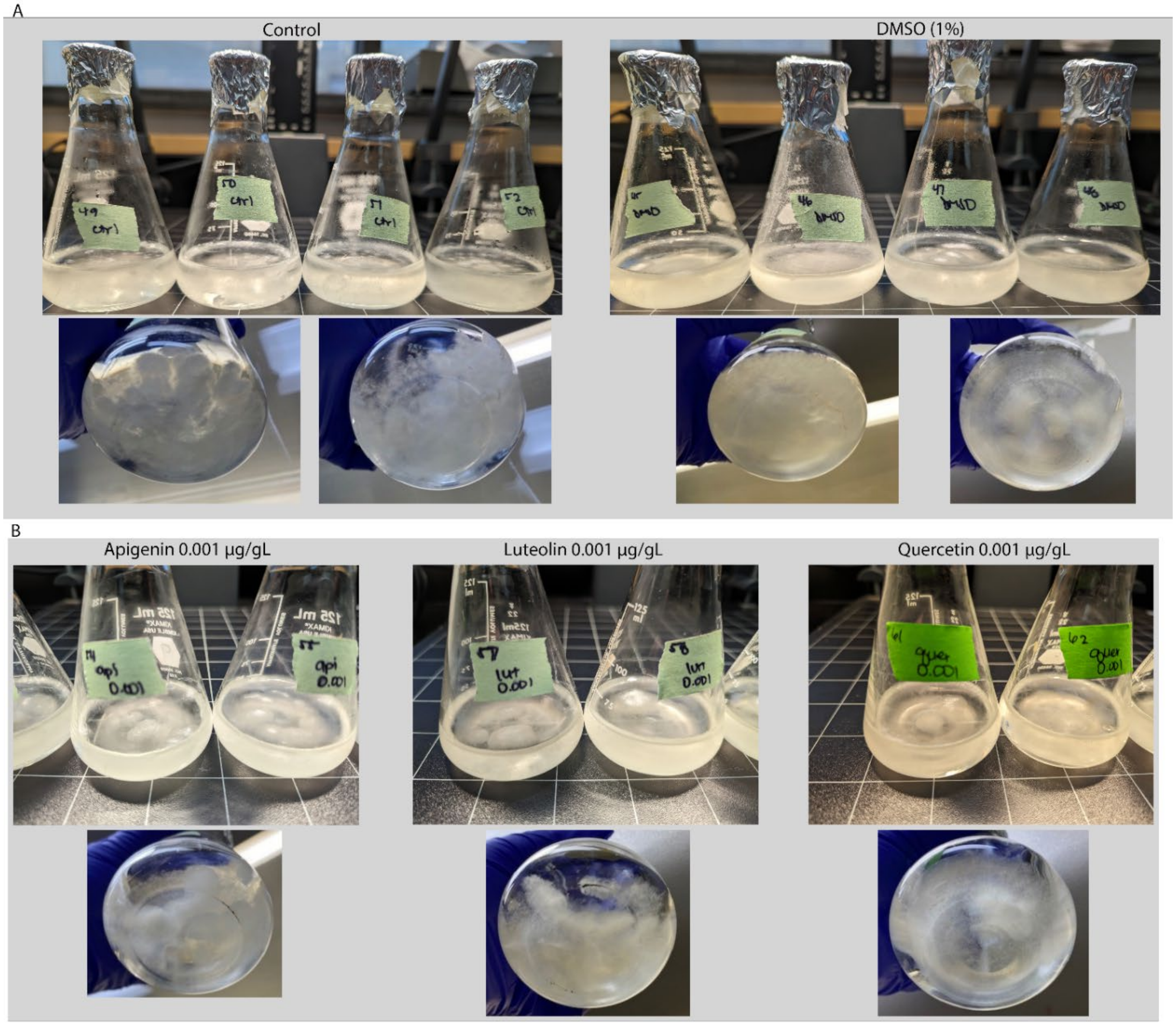

Figure S4. Set up of the bioassay of *A. flavus* 3357 after 72 h incubation in PDB under static conditions. Biological assays performed with AF3357 strain exposed to apigenin, luteolin, and quercetin at A) 0 (1% DMSO and control no-DMSO), and B) 0.001 µg/µL concentrations. N = 4 per treatment.

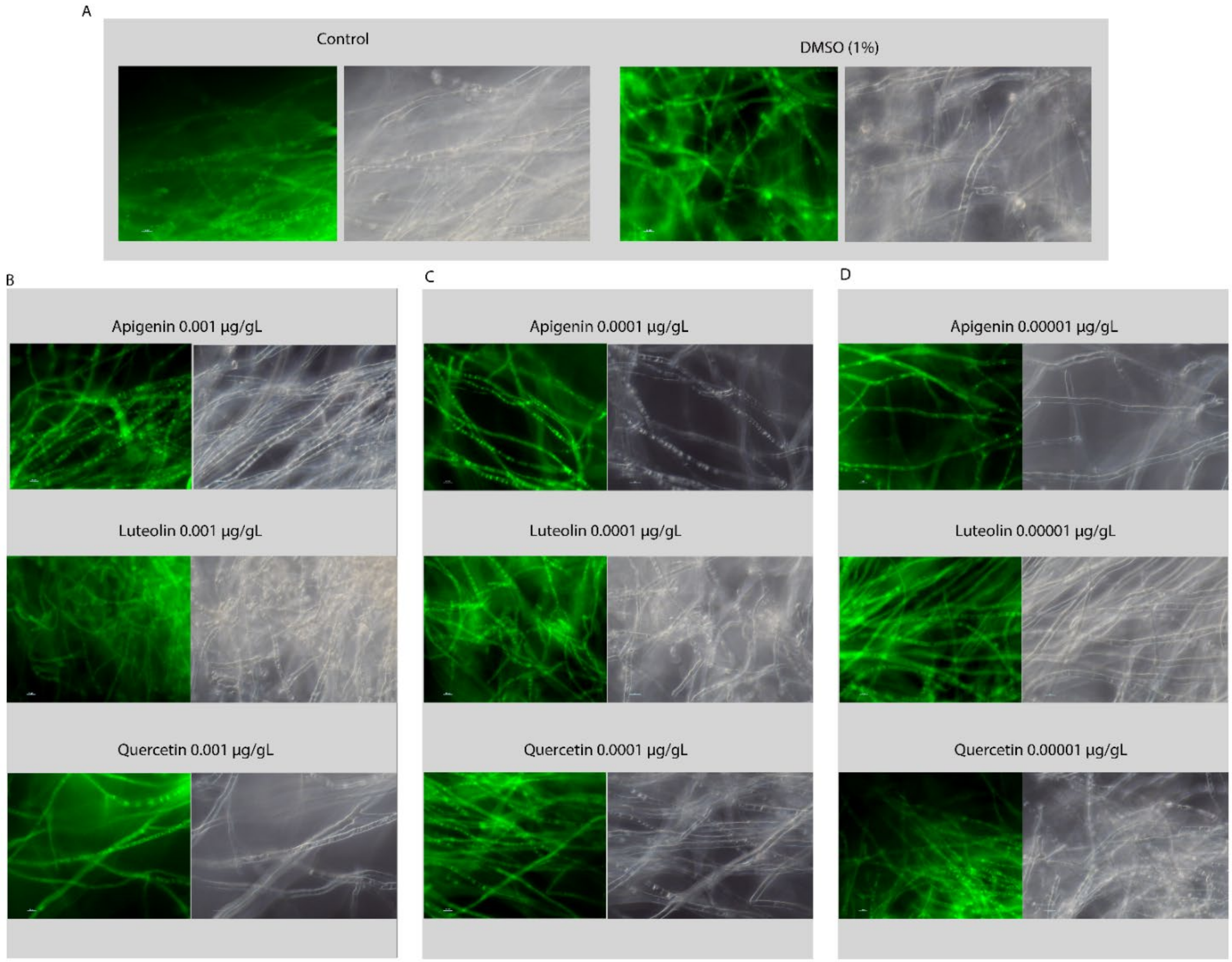

Figure S5. Flavonoids localize outside and inside fungal mycelia. DPBA assay visualized with fluorescence microscopy from AF3357 after 72 h incubation in PDB under shaking conditions. Biological assays performed with AF3357 strain exposed to apigenin, luteolin, and quercetin at A) 0 (1% DMSO and control no-DMSO), and B) 0.001 µg/µL concentrations. White and blue bars represent the scale of magnification. The scale bars are all set to represent 10µm.

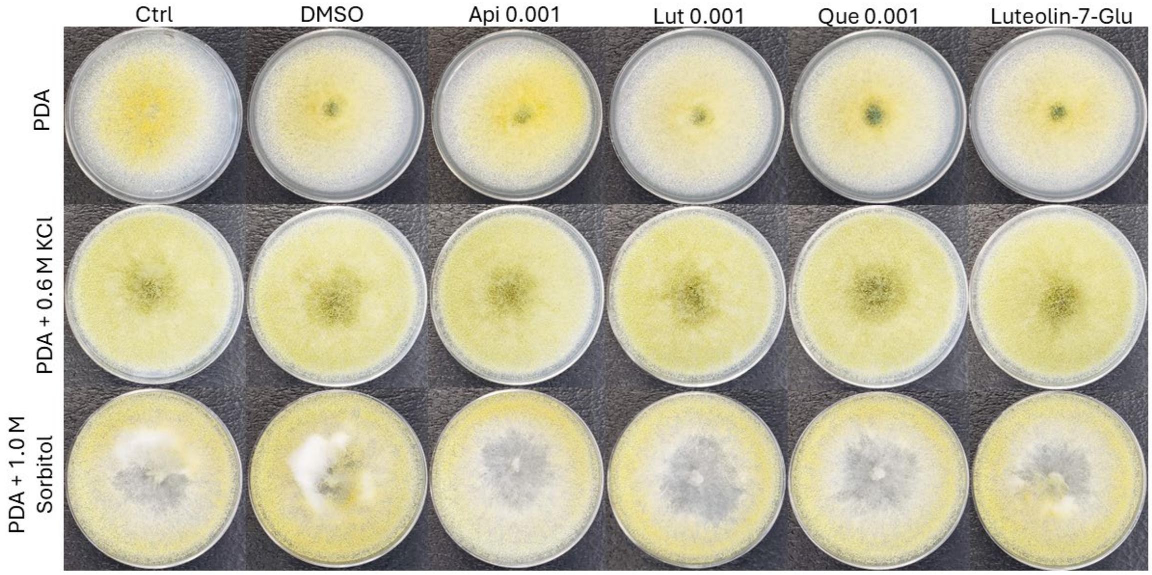

Figure S6. Exposure to osmotic stabilizers did not reveal obvious flavonoid induced cell wall defects in *A. flavus* mycelium. In order to determine if flavonoid treated *A. flavus* mycelium harbored cell wall defects, the AF3357 strain was first grown in the presence and absence of various flavonoid treatments prior to shifting the mycelium on PDA medium and PDA medium supplemented with 0.6 M KCl and 1 M Sorbitol. The cultures were allowed to incubate for 72 h at 31°C prior to observing the cultures for obvious morphological defects and photographing, the experiment was carried out in triplicate.

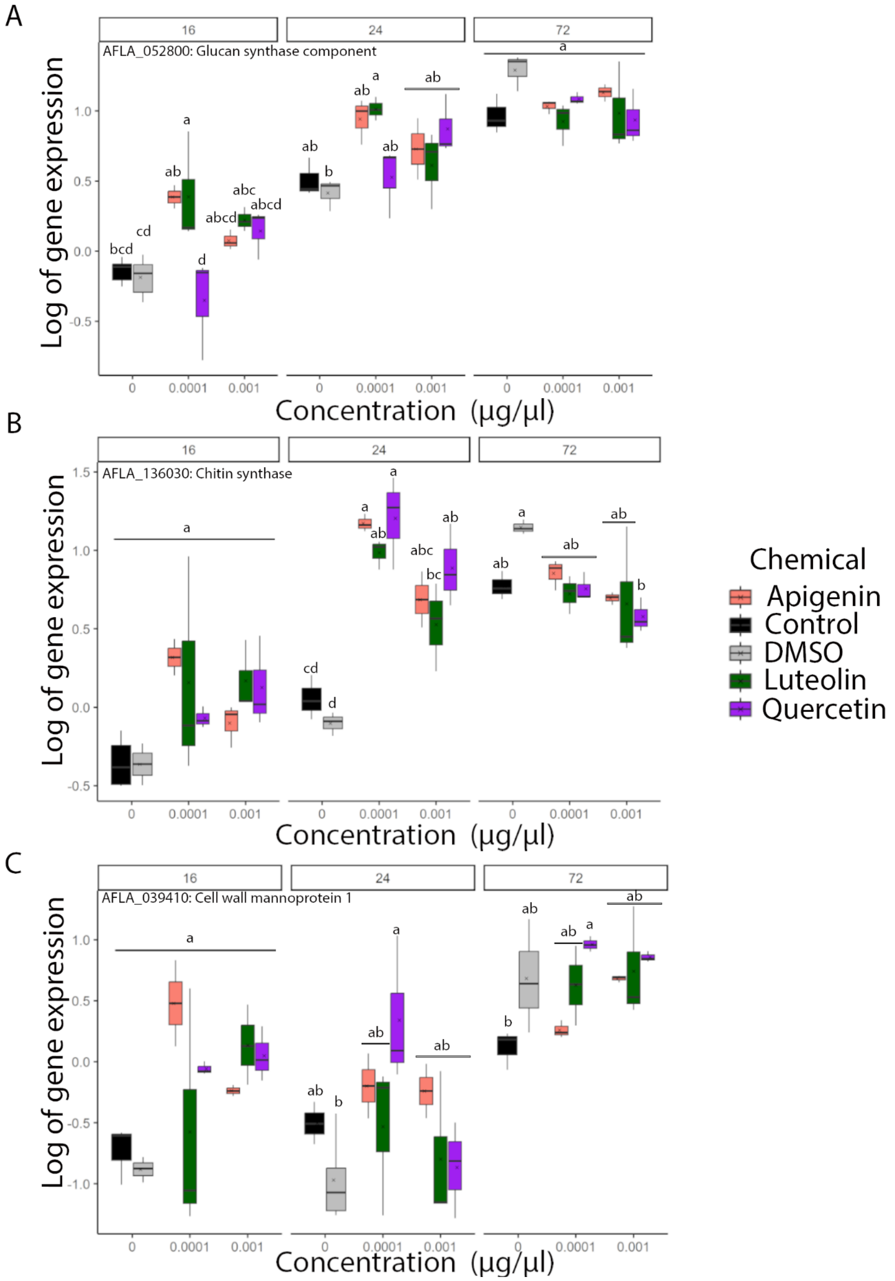

Figure S7. Effect of flavonoids on gene expression of cell wall biosynthesis- related genes from *A. flavus* 3357 at 16, 24 and 72h. Gene expression of A) *AFLA_052800* (Glucan synthase component), B) *AFLA_136030* (Chitin synthase) and C) *AFLA_039410* (Cell wall mannoprotein 1) genes in biological assays performed with AF3357 strain exposed to apigenin, luteolin, and quercetin at 0 (1% DMSO and control no-DMSO), 0.0001, and 0.001 µg/µL concentrations. Key color legend represents chemicals used; salmon: apigenin, green: luteolin, purple: quercetin, black: control (0% DMSO) and gray: DMSO (1% DMSO). Different letters over box plots represent statistically significant differences according to Tukey HSD assay ($p < 0.05$) for gene expression performed separately by day. Box-plot whiskers depict the maximum (25th - 1.5 * interquartile range "IQR") and minimum (75th percentile + 1.5 * interquartile range (IQR)), and the box depicts median, first (25th percentile) and third (75th

percentile) quantiles distribution. N = 3 per treatment.

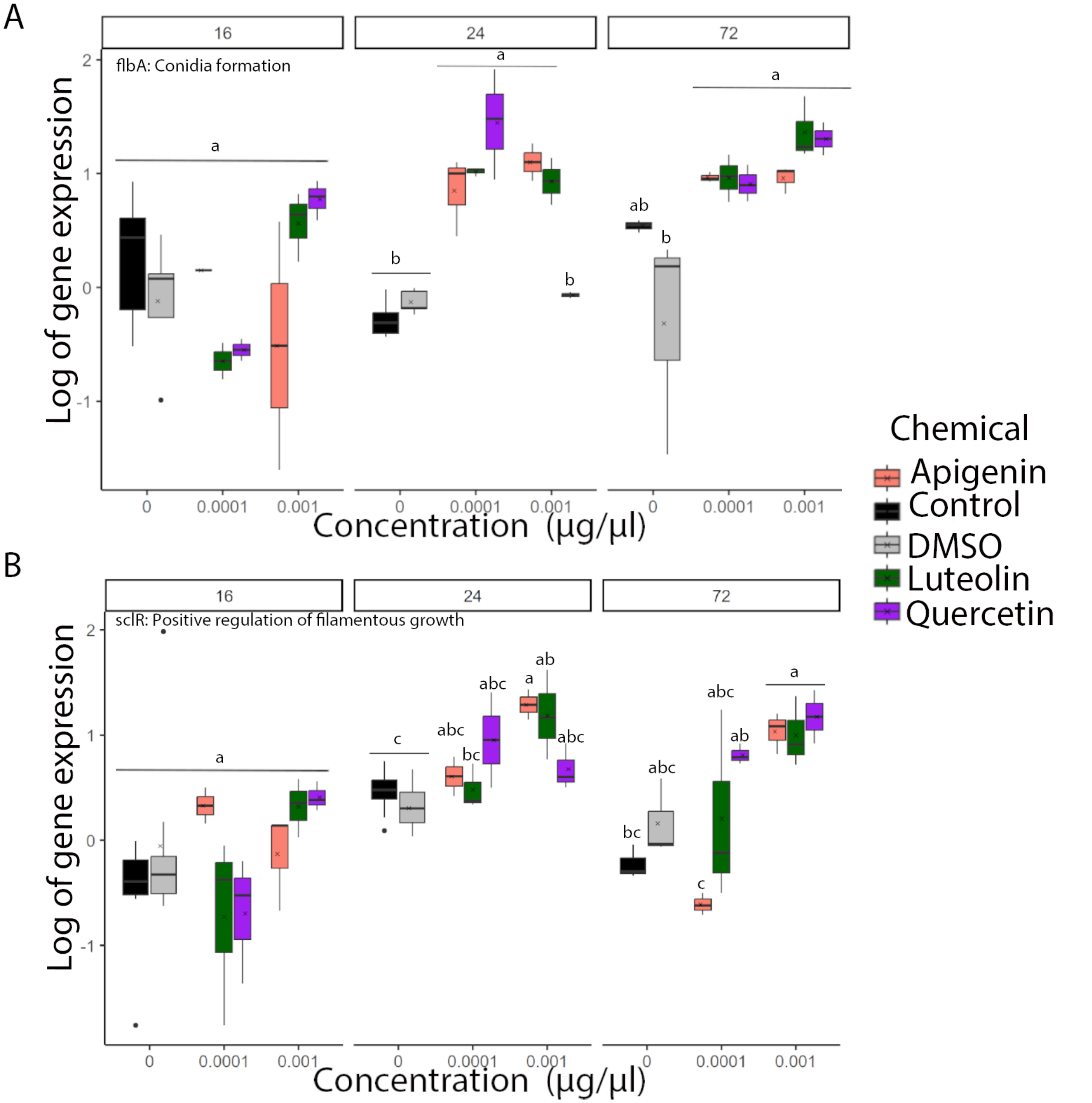

Figure S8. Effect of flavonoids on expression of fungal development-related genes from *A. flavus* 3357 at 16 and 24 h. Gene expression of A) *flbA* and B) *sclR* genes in biological assays performed with AF3357 strain exposed to apigenin, luteolin, and quercetin at 0 (1% DMSO and control no-DMSO), 0.0001, and 0.001 μg/μL concentrations. Key color legend represents chemicals used; salmon: apigenin, green: luteolin, purple: quercetin, black: control (0% DMSO) and gray: DMSO (1% DMSO). Different letters over box plots represent statistically significant differences according to Tukey HSD assay ($p < 0.05$) for gene expression performed separately by day. Box-plot whiskers depict the maximum (25th - 1.5 * interquartile range “IQR”) and minimum (75th percentile + 1.5 * interquartile range (IQR)), and the box depicts median, first (25th percentile) and third (75th percentile) quantiles distribution. N = 3 per treatment.

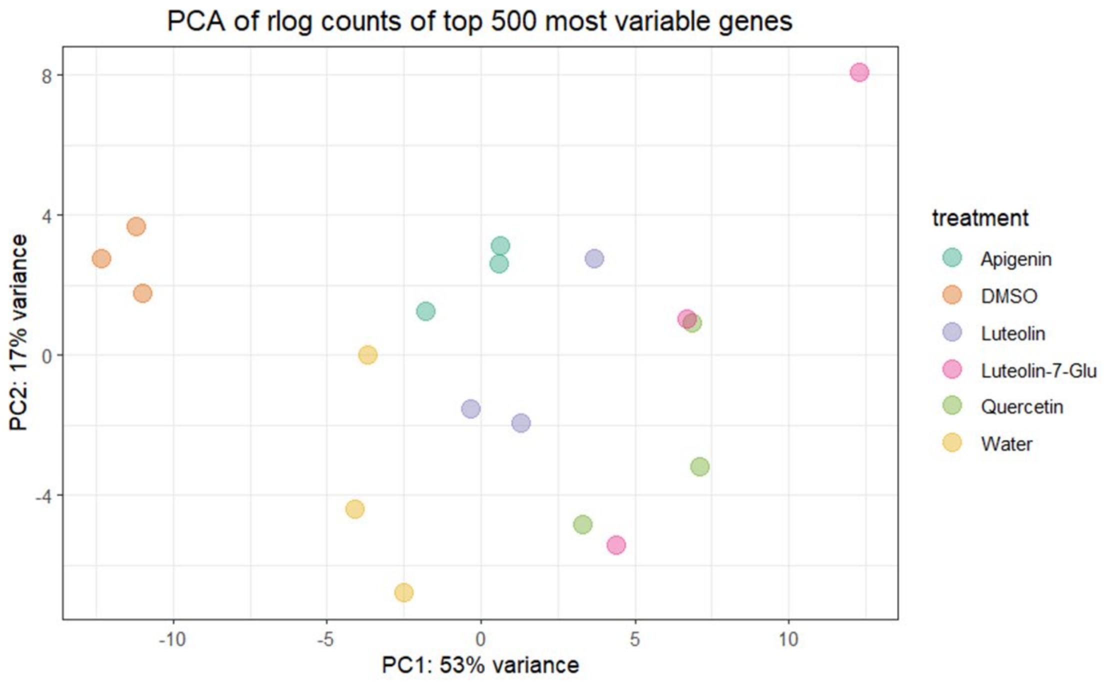

Figure S9. Sample variation of RNA-seq experiment. Principal component analysis of gene expression sequencing data from AF3357 treated with 0.001 μg/μL of flavonoids (apigenin, luteolin, luteolin-7-glucoside and quercetin), 1% DMSO or buffer control.

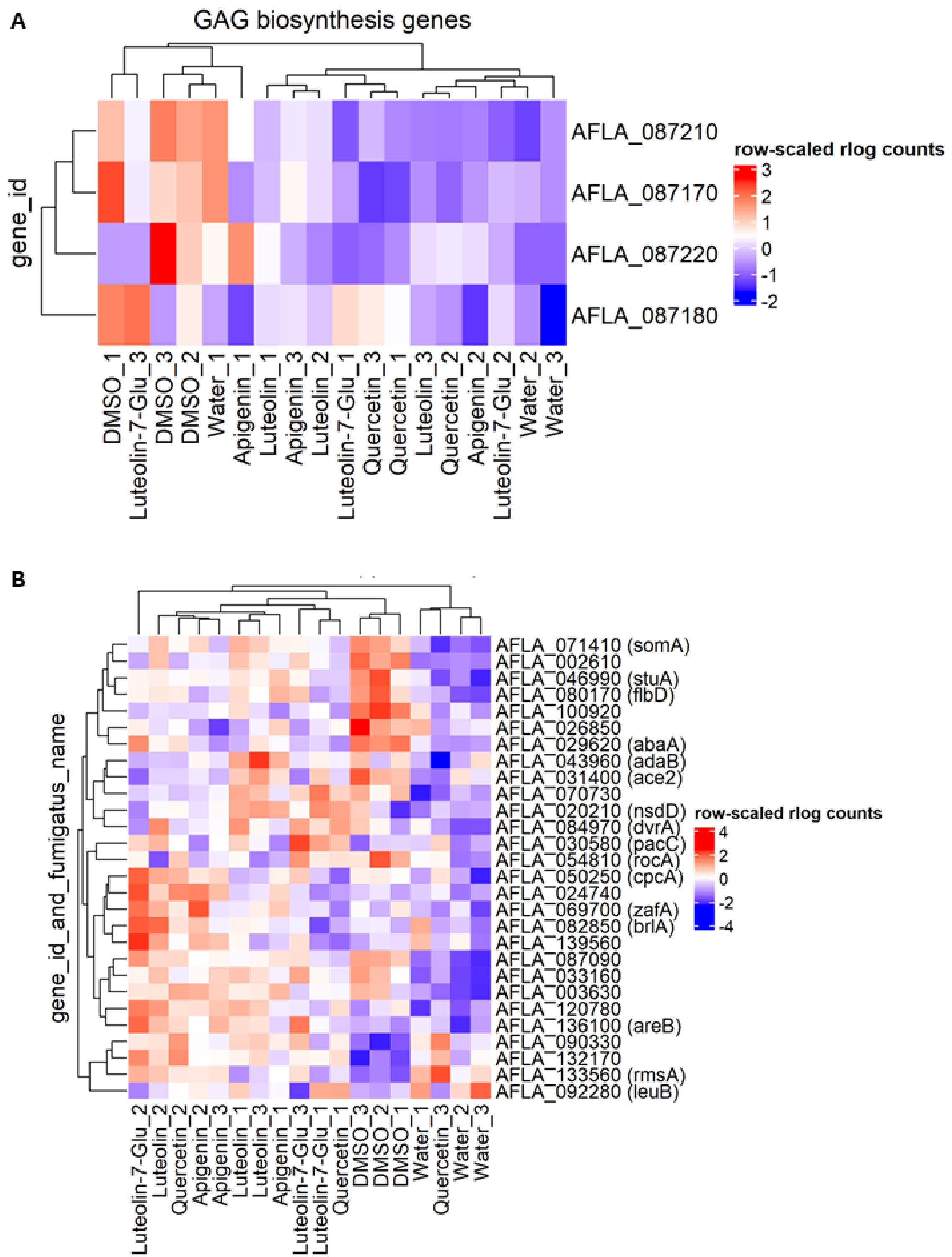

Figure S10. Effect of flavonoids on GAG biosynthesis and signaling pathways. Hierarchical heatmap genes expression from AF3357 treated with 0.001 µg/µL of flavonoids (apigenin, luteolin, luteolin-7-glucoside (Lut_7-Glu) and quercetin), 1% DMSO or buffer control. Putative genes in *A. flavus* that are in the A) GAG biosynthesis pathway and B) GAG signaling pathways.
