# Supplementary figures and images for: Investigating the Impact of Flavonoids on Aspergillus flavus: Insights into Cell Wall Damage and Biofilms
Source: J Fungi (Basel). 2024 Sep 23;10(9):665. doi: 10.3390/jof10090665 (PMC11433479; doi:10.3390/jof10090665)

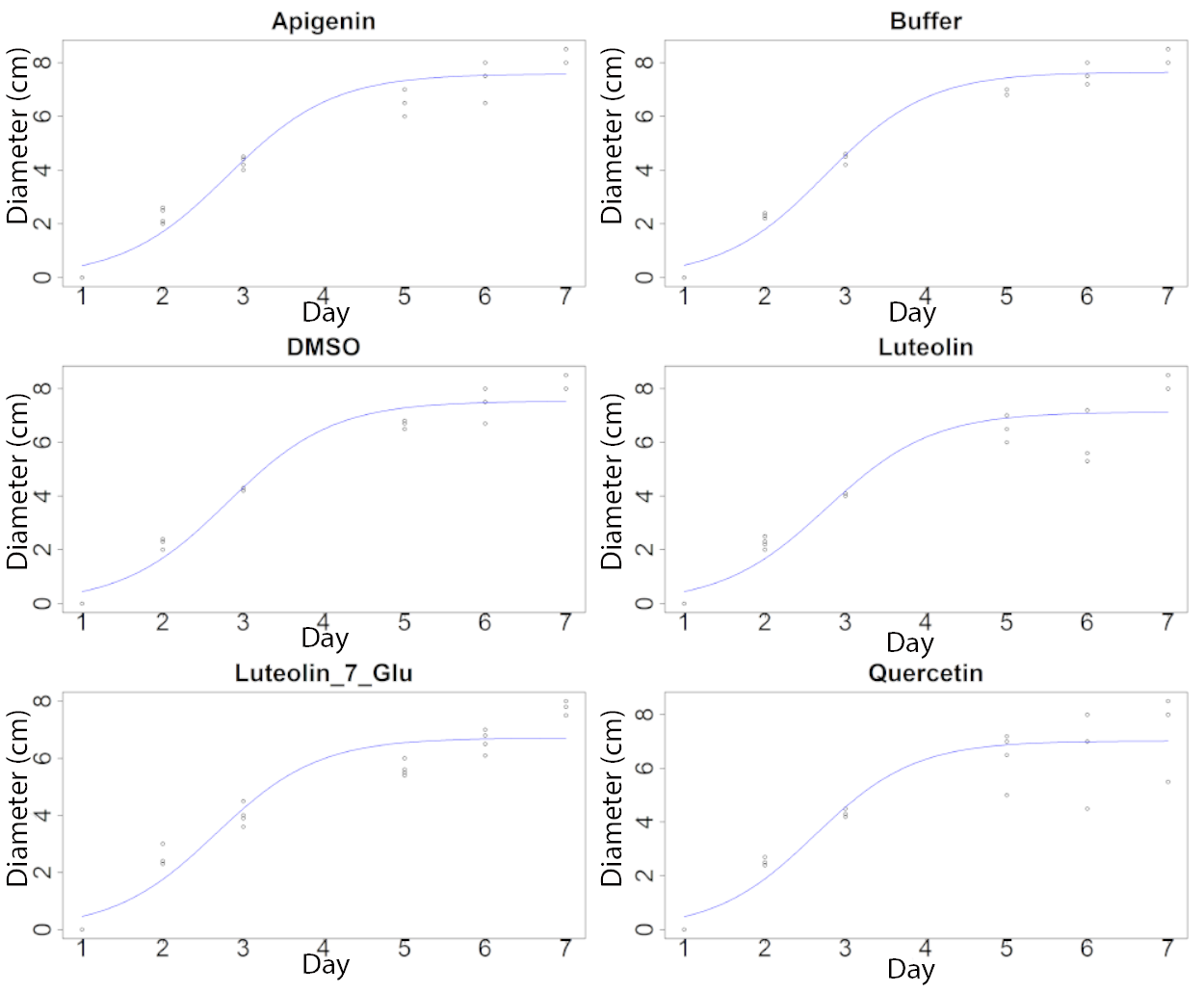

Supplement: Supplementary file 1 [file jof-10-00665-s001.zip › FigureS1.png]

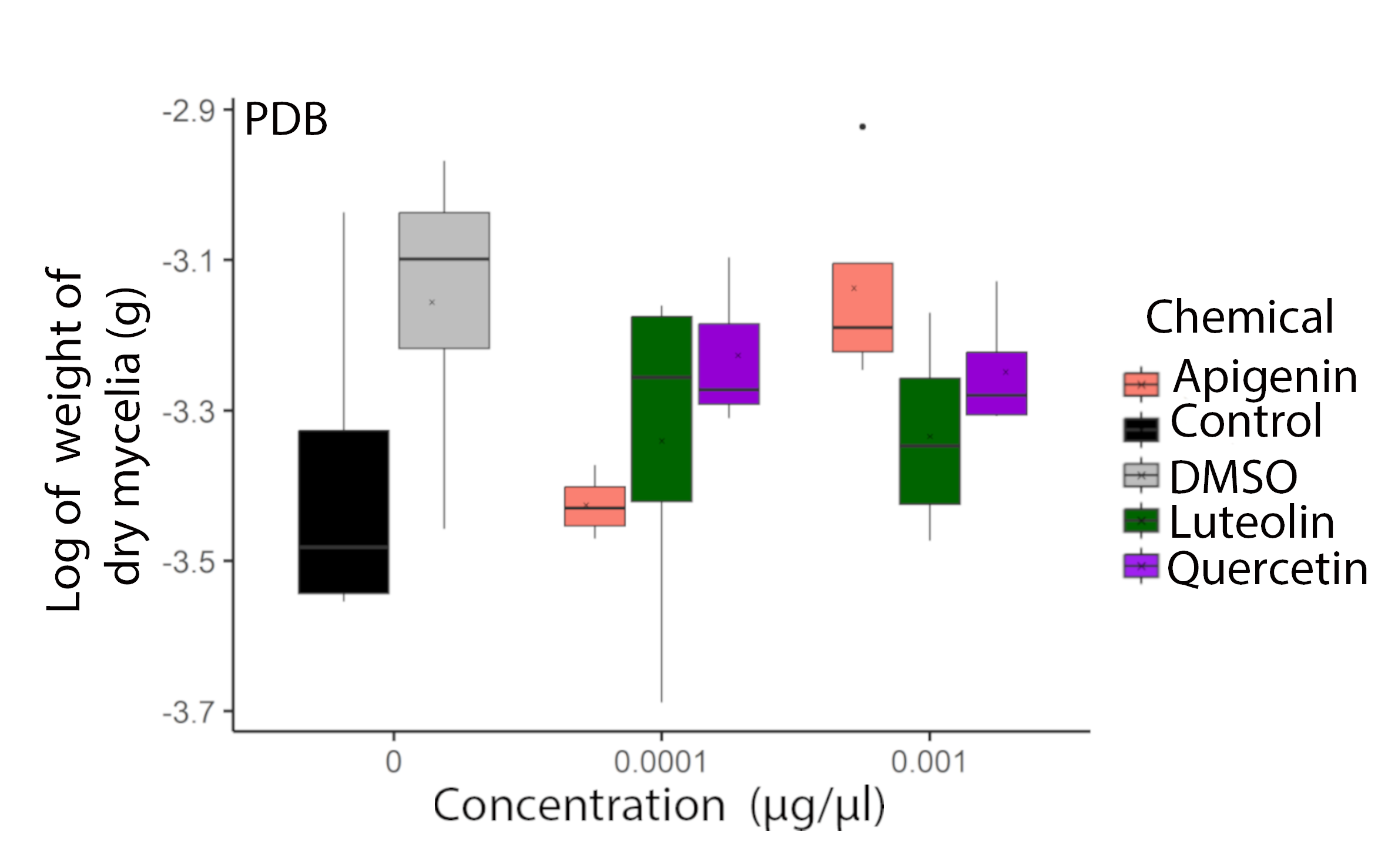

Supplement: Supplementary file 1 [file jof-10-00665-s001.zip › FigureS2.png]

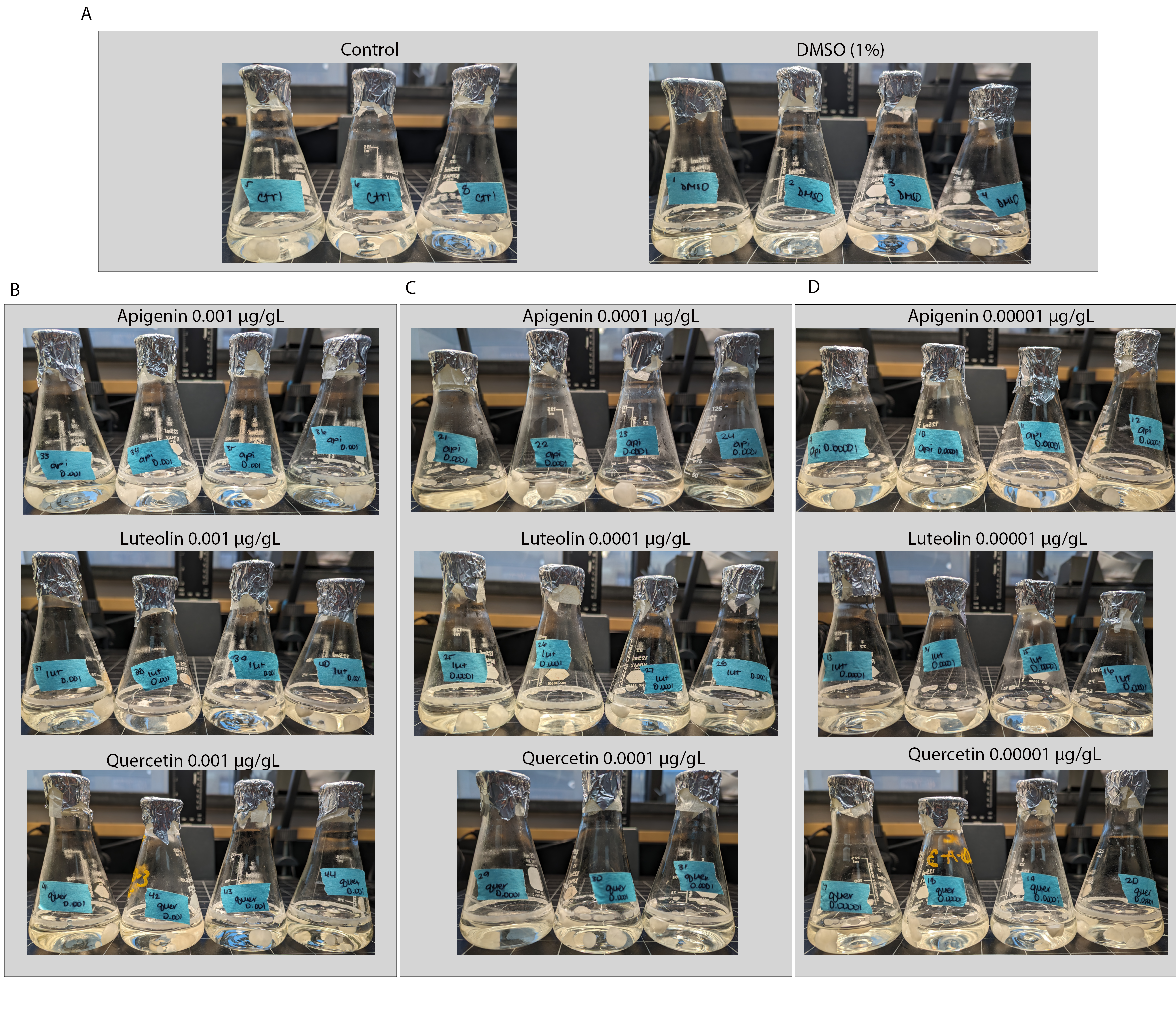

Supplement: Supplementary file 1 [file jof-10-00665-s001.zip › FigureS3.png]

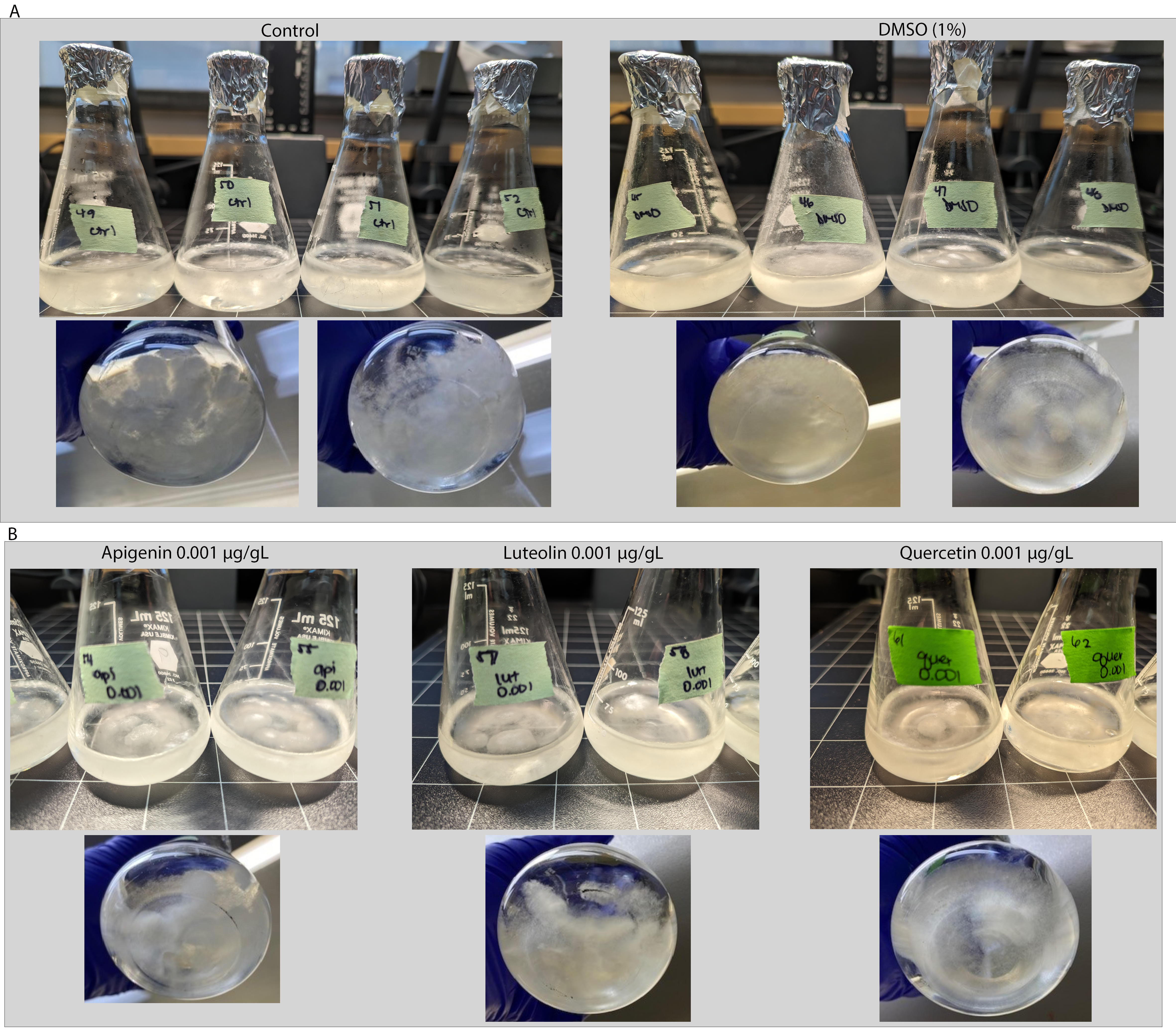

Supplement: Supplementary file 1 [file jof-10-00665-s001.zip › FigureS4.png]

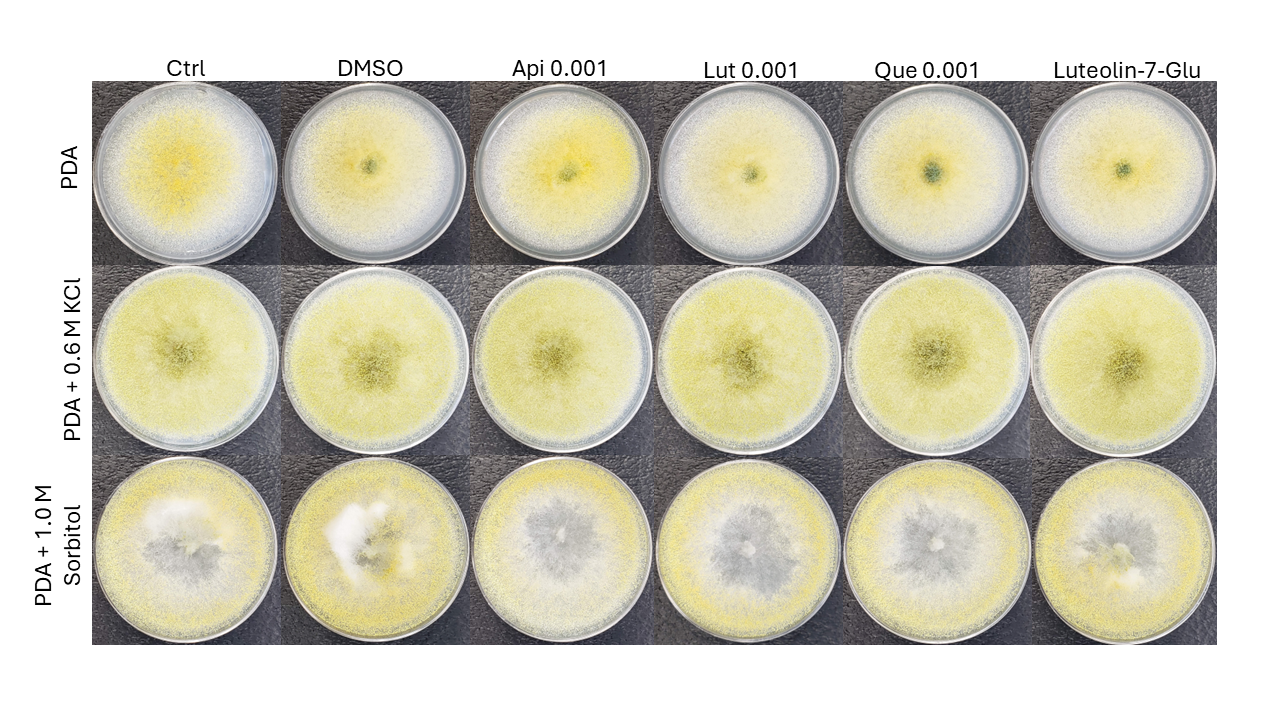

Supplement: Supplementary file 1 [file jof-10-00665-s001.zip › FigureS6.png]

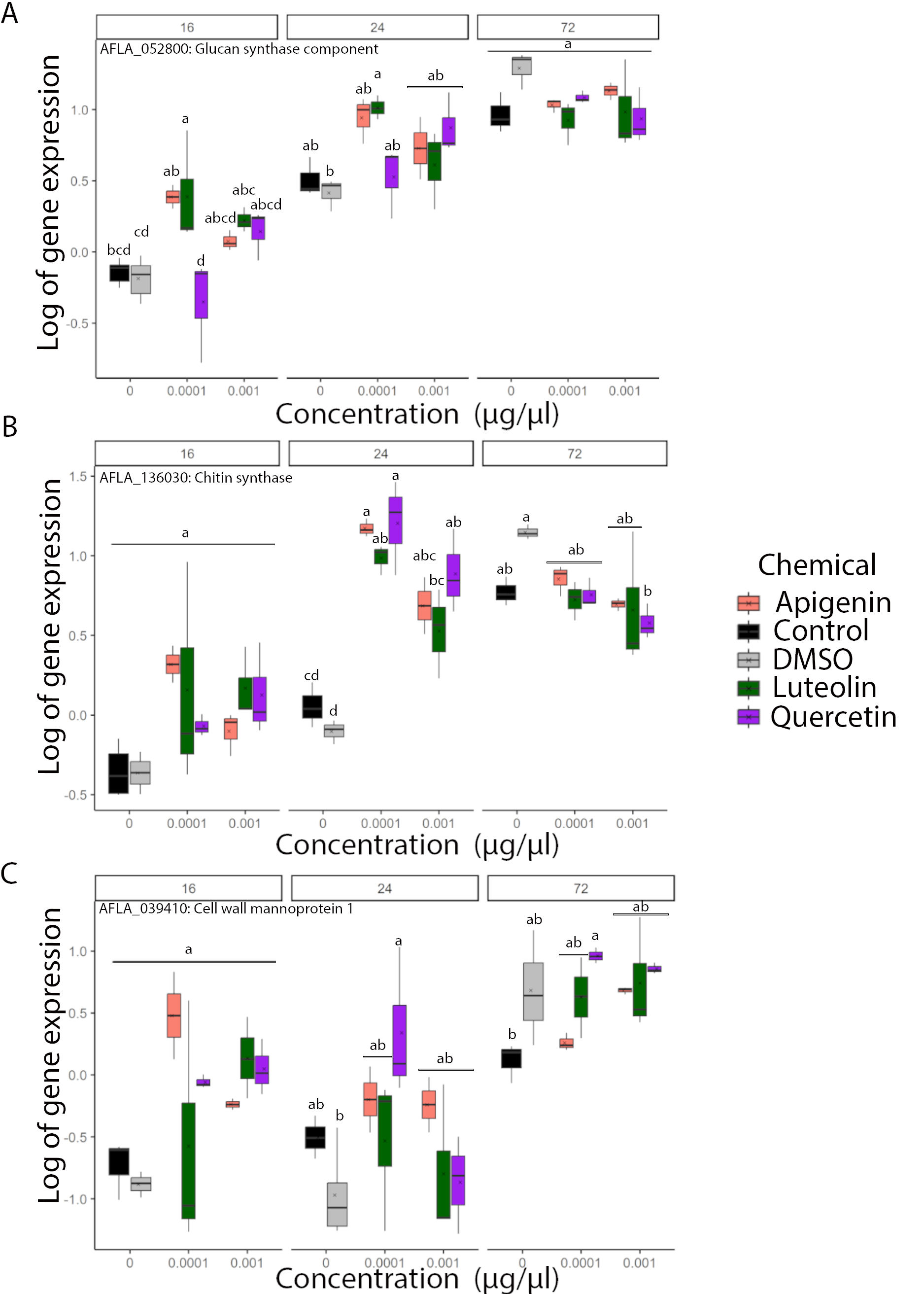

Supplement: Supplementary file 1 [file jof-10-00665-s001.zip › FigureS7.png]

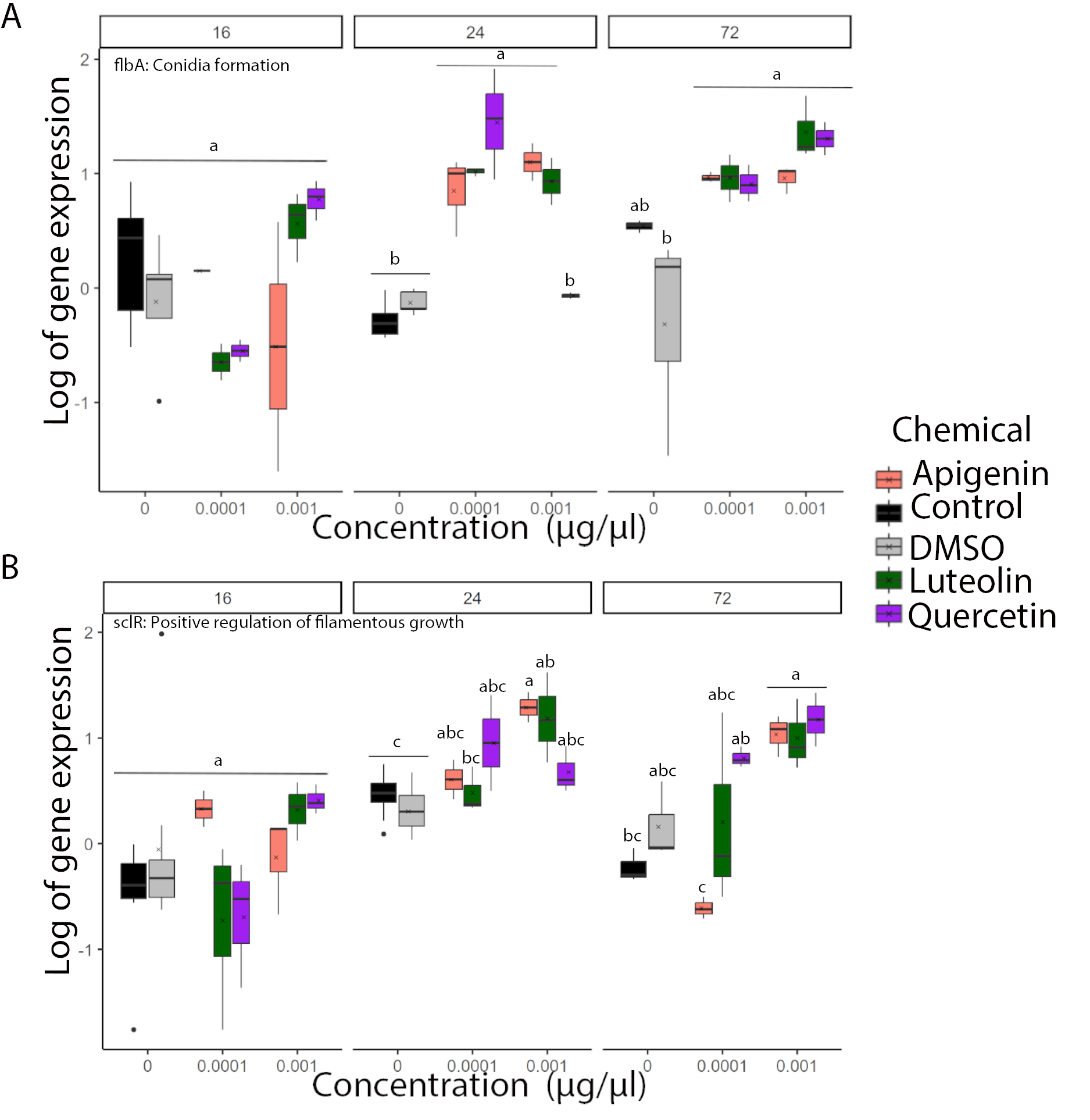

Supplement: Supplementary file 1 [file jof-10-00665-s001.zip › FigureS8.png]

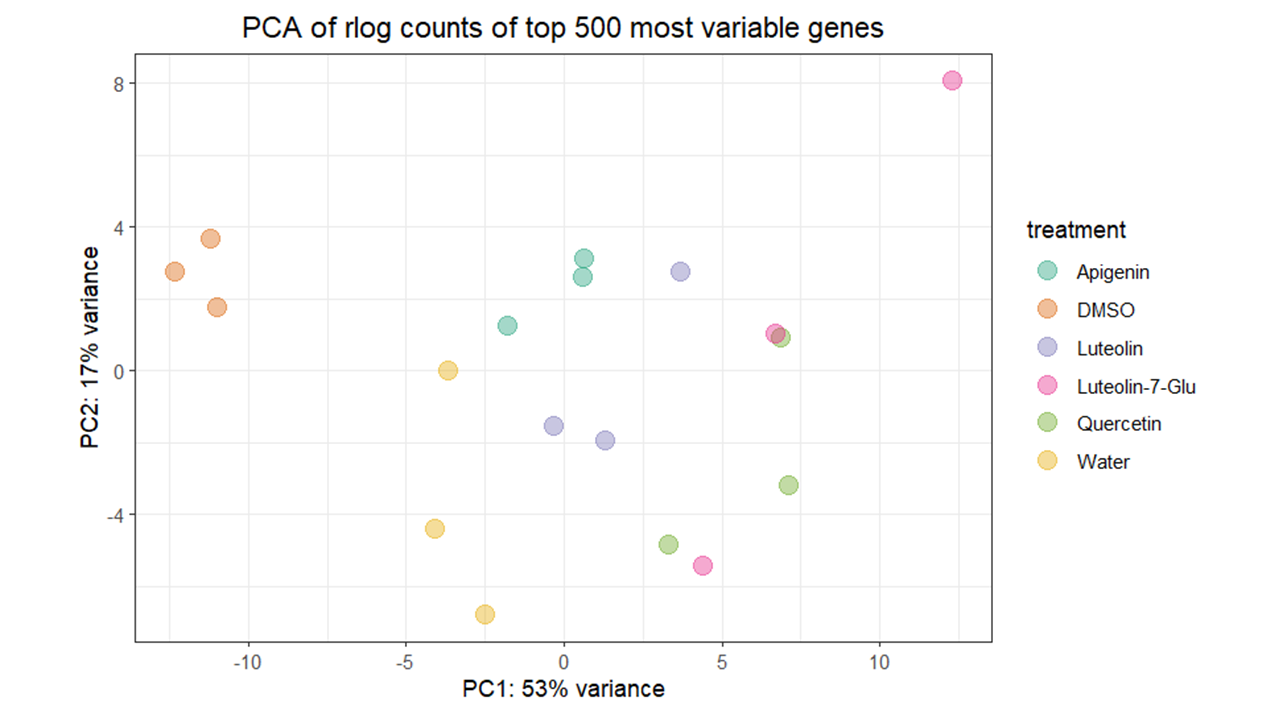

Supplement: Supplementary file 1 [file jof-10-00665-s001.zip › FigureS9.png]

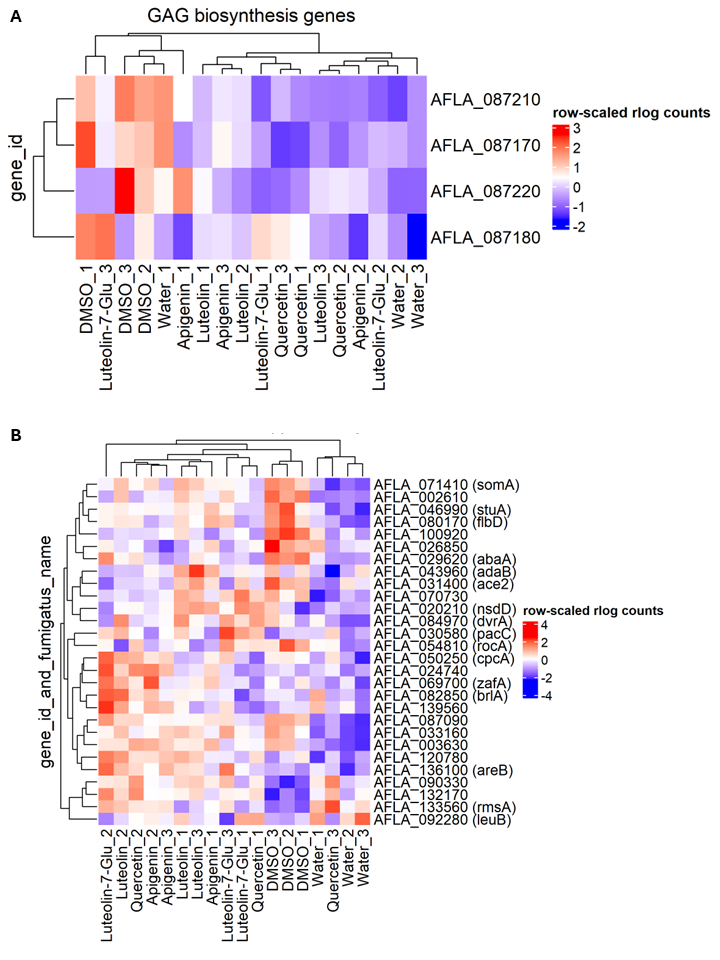

Supplement: Supplementary file 1 [file jof-10-00665-s001.zip › FigureS10.png]
